# Plant growth forms dictate adaptations to the local climate

**DOI:** 10.3389/fpls.2022.1023595

**Published:** 2022-11-21

**Authors:** Patrícia dos Santos, Miguel Ângelo Brilhante, Thibaud F. E. Messerschmid, Helena Cristina Serrano, Gudrun Kadereit, Cristina Branquinho, Jurriaan M. de Vos

**Affiliations:** ^1^ Centre for Ecology, Evolution and Environmental Changes (cE3c) & Global Change and Sustainability Institute (CHANGE), Faculdade de Ciências, Universidade de Lisboa, Lisboa, Portugal; ^2^ Department of Environmental Sciences – Botany, University of Basel, Basel, Switzerland; ^3^ Linking Landscape, Environment, Agriculture and Food (LEAF), Instituto Superior de Agronomia (ISA), Universidade de Lisboa, Lisbon, Portugal; ^4^ Botanischer Garten München-Nymphenburg und Botanischen Staatssammlung, Staatliche Naturwissenschaftliche Sammlungen Bayerns, Munich, Germany; ^5^ Prinzessin Therese von Bayern Lehrstuhl für Systematik, Biodiversität & Evolution der Pflanzen, Ludwig-Maximilans-Universität München, Munich, Germany

**Keywords:** adaptive radiation, *Aeonium*, Canary Islands, Crassulaceae, ecological adaptations, trait evolution

## Abstract

Adaptive radiation is a significant driver of biodiversity. Primarily studied in animal systems, mechanisms that trigger adaptive radiations remain poorly understood in plants. A frequently claimed indicator of adaptive radiation in plants is growth form diversity when tied to the occupation of different habitats. However, it remains obscure whether morphological adaptations manifest as growth form diversity per se or as its constituent traits. We use the classic *Aeonium* radiation from the Canary Islands to ask whether adaptation across climatic space is structured by growth form evolution. Using morphological sampling with site-associated climate in a phylogenetic context, we find that growth forms dictate adaptations to the local environment. Furthermore, we demonstrate that the response of specific traits to analogous environments is antagonistic when growth forms are different. This finding suggests for the first time that growth forms represent particular ecological functions, allowing the co-occurrence of closely related species, being a product of divergent selection during evolution in sympatry.

## Introduction

Adaptive radiations are the source of much of the biodiversity on Earth, resulting from the evolution of phenotypic diversity in response to ecological shifts within a rapidly multiplying lineage (Grant, 1981; Schluter, 2000; Givnish, 2015). Examples such as the Darwin finches from the Galapagos Islands, the *Anolis* lizards of the Greater Antilles and the cichlid fishes from East African lakes demonstrate how adaptations to the local environment result in a rapid accumulation of morphological and ecological diversity (Grant, 1981; Losos et al., 1998; Schluter, 2000; Emerson, 2002; Grant and Grant, 2008; Losos and Ricklefs, 2009; Feiner et al., 2021; Ronco et al., 2021).

In plants, adaptive radiations remain relatively poorly understood, as the particular associations between morphological and ecological diversity of species remain elusive (Schenk, 2021). Growth forms have been proposed as plant adaptations to the environment under evolutionary radiations to facilitate the colonization of different habitats (Lems, 1960; Rowe and Speck, 2005; Dunbar-Co et al., 2008; Schenk et al., 2008; Anest et al., 2021). Plant growth forms are frequently recognized as suits of individual plant traits that jointly implement some ecological role and, consequently, are generally interpreted as adaptations to the environment, such as high winds, erratic rainfall regimes, grazing, or frost (Rowe and Speck, 2005; Santiago and Wright, 2007; Schenk et al., 2008; Gardiner et al., 2016; Anest et al., 2021). While some authors distinguish growth form (the architectural type) and life form (the phenotypic result of that architecture with the environment) (Rauh, 1978), growth form disparity is consistently prevalent in well-known putative adaptive radiations, such as the Hawaiian silversword alliance (Asteraceae) (Carr, 1987; Robichaux et al., 1990; Blonder et al., 2016; Landis et al., 2018), the Hawaiian lobeliads (Campanulaceae) (Givnish et al., 2004; Givnish et al., 2008; Givnish, 2010) or the Andean *Lupinus* (Fabaceae) (Drummond, 2008; Drummond et al., 2012; Hughes and Atchison, 2015; Nevado et al., 2016; Contreras-Ortiz et al., 2018). Clear examples of recurrent evolution of growth forms in particular environments include small-stature shrubs on tropical mountains (Nürk et al., 2013; Gehrke et al., 2016), succulent plants in seasonally dry environments (Ringelberg et al., 2020) or cushion plants in the alpine biome (Körner et al., 2021), where structural aspects of plant form vary in association with other traits (e.g., leaf size, number, and thickness) (Prusinkiewicz et al., 2007; Harder and Prusinkiewicz, 2013). However, growth form diversity can also arise through the evolution of divergent ecological roles (Dunbar-Co et al., 2008), e.g., perennial herbs, shrubs, trees, and lianas may co-occur within a forest. This suggests that growth form disparity may arise from divergent selection resulting from complex biotic and abiotic interactions. Overall, the role of growth form diversity in plant adaptive radiations remains largely untested, as phenotype-environment relations of growth forms remain mostly undocumented (Schluter, 2000; Anest et al., 2021).

**Figure 1:**
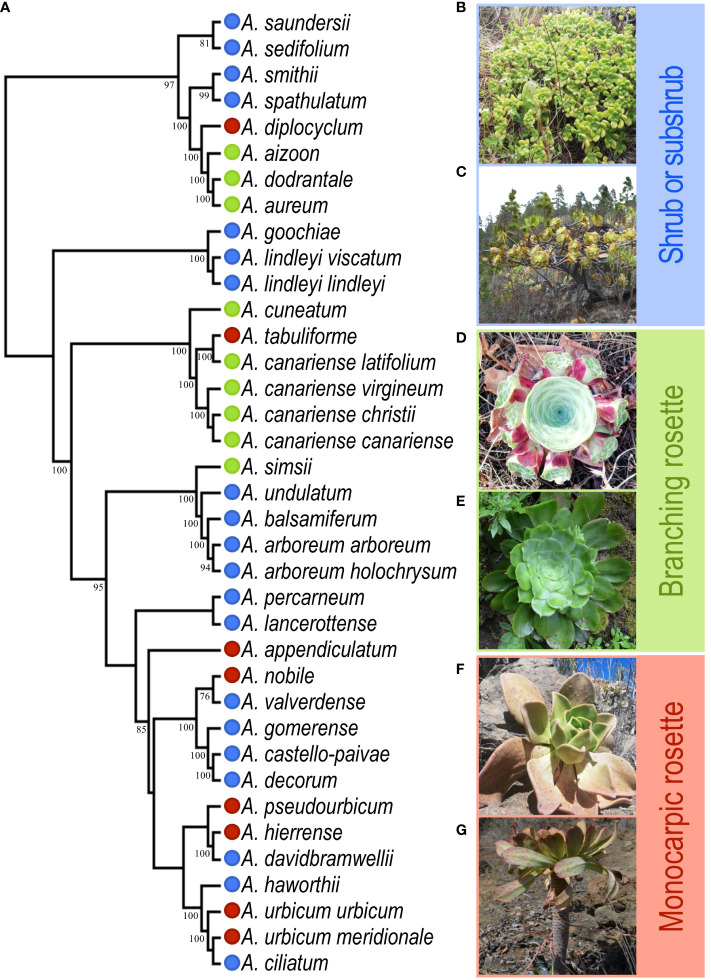
Phylogeny and growth form diversity in *Aeonium*. **(A)** Adapted phylogeny from Messerschmid et al. (accepted pending revision) to include only the taxa used in this study; numbers below branches indicate bootstrap values >75. Growth forms are represented by colour code: Shrub and subshrub (SS) in blue, ground-branching rosettes (BR) in green and monocarpic rosettes (MR) in red. **(B)** *A. lindleyi* ssp. *lindleyi*; **(C)** *A. arboreum* ssp. *holochrysum*; **(D)** *A. aureum*; **(E)** *A. cuneatum*; **(F)** *A. nobile*; **(G)** *A. urbicum* ssp. *urbicum*.

*Aeonium* (Crassulaceae) diversified on the Canary Islands (~8 Mya) throughout a multitude of habitats, resulting in the most speciose Canarian plant genus (Kim et al., 2008; Schenk, 2021). This diversification resulted in a spectacular array of growth forms; however, its classification remains without consensus despite gathering the attention of several studies in the past (Lems, 1960; Mes and ‘T Hart, 1996; Mort et al., 2007). Here, we used a simple classification based on the three general growth forms: ground-branching rosette plants (BR), monocarpic rosettes (MR) and large shrubs to sub-shrub species (SS; **Figure 1**).

We empirically test the hypothesis that growth forms provide selective advantages to abiotic factors by determining the climatic and phenotypic dimensions of the *Aeonium* radiation in a phylogenetic context. Specifically, we extensively sampled 37 Canarian endemics to (1) quantify the fraction of extant climate space occupied by the genus in the Canary Islands; (2) define the climatic dimensions of growth forms; (3) determine whether growth form effectively capture morphological diversity; and (4) test the association of growth forms and individual traits to climate. This work reveals the fundamental ecological and evolutionary roles of growth form diversity in plant adaptive radiations.

## Material and methods

### Study system and data collection

**Figure 2:**
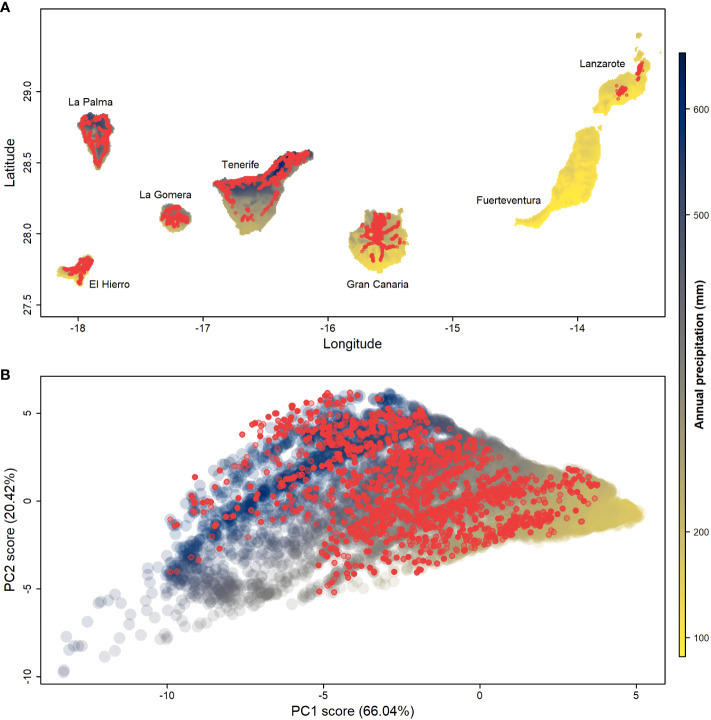
Climate space on the Canary Islands and climatic occupation of *Aeonium* on the Canary Islands. **(A)** Map of the Canary Islands colored according to the annual precipitation gradient; red dots represent *Aeonium* sampling sites used to calculate the climatic occupation on the islands; **(B)** Principal components analysis (PCA) of the climatic range throughout the Canary Islands. Dots colored by the annual precipitation gradient are random points extracted from the map **(A)** to represent the overall climatic space; red dots represent the *Aeonium* spatial sampling sites extracted from **(A)**. PCA loadings can be found in **Supplementary Table 4**.

The Canary Islands is the largest archipelago of Macaronesia, located in the North Atlantic Ocean (27-29°N, 13-18°W), about 100 km off the coast of the Western Sahara Desert (**Figure 2A**). The ages of the seven volcanic islands range from ca. 1 Mya in the West to ca. 24 Mya in the East (Day et al., 1997; Hoernle and Carracedo, 2009), representing a cline in island erosion and concomitantly geomorphological complexity. Moreover, tradewinds cause intense precipitation and temperature gradients, both within the steep North-Western Islands that exhibit extreme differences in elevation and between humid northern and dry southern sectors, and across the archipelago. The islands’ immense climatic variation is typically divided into five major habitat types: sub-tropical laurel forest, pine forest, thermophile shrubland, xerophytic scrubland, and alpine (Fernández-Palacios et al., 2008; Bañares-Baudet, 2015). With an estimated 35 to 45% of plant endemism among the ~1300 native plant species, along with the diverse island ages and breath of climatic conditions, the Canary Islands are considered a classic “evolutionary laboratory” (Emerson, 2002; Whittaker et al., 2017).

*Aeonium* (Crassulaceae) is a rosette-forming succulent genus with several growth forms described (**Figure 1**) (Lems, 1960; Bañares-Baudet, 2015). It is a monophyletic genus of 44 taxa (34 species and ten subspecies), almost all endemic to the Canary Islands, except for six phylogenetically deeply nested species from Morocco, Madeira, Cape Verde, and East Africa (Liu, 1989). We only considered the Canary Islands representatives for this study. To collect morphological and spatial data, we visited all islands with endemic species between April 2018 and August 2020 (i.e., excluding Fuerteventura, whose only species, *A. balsamiferum*, was sampled in Lanzarote).

Growth forms were quantified using multivariate and univariate approaches, aiming for five plants from each of three populations with a large number of healthy mature individuals per taxon (**Supplementary Table 1**). Accordingly, we sampled all 37 Canary-island taxa (27 species and all ten subspecies; varieties were not considered in the sampling; **Figure 1**). Considering the rarity of some of these endemisms, including nine endangered species (Bañares-Baudet, 2015), the minimum of three populations per taxon was not always achieved (**Supplementary Table 1**). Two species were excluded from this study: *A. mascaense* due to being extinct in the wild (Bañares-Baudet, 2015) and *A. volkerii* because only one healthy individual was found in the field. We followed the most recent classification for species identification in the field (Bañares-Baudet, 2015). In each population, we registered the geolocation at its approximate centroid using a high-sensitivity Global Positioning System receiver (GPS 72H, Garmin, Taiwan; positional accuracy ca. 5 m). The univariate data used a classic scheme of three primary growth forms: shrubs and subshrubs (SS) for branched taxa with stems, branching rosette (BR) for stemless branched taxa (branching at or below the surface through stolons, forming prostrate rosettes), and monocarpic rosette (MR) for unbranched taxa. These growth forms capture two essential traits: rosette number (1 in MR and > 1 in others) and the branching pattern (elongated above-surface stems in SS and underground in stemless-BR). Though this approach was simply implemented in the field, in rare situations, individuals showed different growth forms than expected according to the species descriptions (e.g., *A. diplocyclum* showed BR growth form in one population from La Palma). In this situation, we classified the growth form of the species overall as described by Bañares-Baudet (2015) for congruence, as MR revealed to be the most common growth form for the species in other populations. The multivariate data captured diversity in plant form more comprehensively through eleven vegetative traits: rosette number, plant height, rosette diameter, maximum plant diameter, minimum plant diameter, leaf length, leaf width, petiole width, leaf thickness, rosette area and photosynthetic area (**Supplementary Table 2**). In total, we sampled 528 plants from 37 species and subspecies in 99 populations distributed along 70 locations for morphology (**Supplementary Table 1**).

To capture the environmental breadth of the radiation, we recorded additional occurrences of all encountered species in an environmentally stratified approach. Specifically, for each island, we determined four climatic quadrants defined by the intersection of mean annual temperature and annual precipitation (warm and wet, warm and dry, cold and wet, and cold and dry). We made sure we searched for *Aeonium* in all four quadrants on each island, recording localities as above. Using this approach, we recorded 7730 localities with *Aeonium*. Note that possible spatial autocorrelation among observations is not relevant in the present study, as we do not consider the density of observations along climatic axes, only their span. For each locality, we extracted the 19 bioclimatic and the monthly potential solar radiation rasters (summed to their yearly value) at a resolution of 30 arcsec from CHELSA V.1.2 (Karger et al., 2017; Karger et al., 2018) (**Supplementary Table 3**). Spatial data handling relied on the package *raster* (Hijmans, 2021) of the statistical computing environment R, version 4.1.0 (R Core Team, 2021).

Phylogenetic information on relations among taxa was extracted from the well-resolved RAxML phylogeny of Messerschmid et al. (accepted pending revision), which was based on a concatenated supermatrix of loci in the length range 320–500 nucleotides obtained from multi-locus long read ddRAD sequence data (Hühn et al., 2022). The phylogeny was then pruned to include only sampled taxa only and made ultrametric using the ape function *chronos* (Paradis, 2013) while scaling the root using the corresponding age estimate of 4.62 Mya (**Figure 1A**).

### Statistical analyses

Statistical analyses were performed using the statistical computing environment R, software version 4.1.0 (R Core Team, 2021). To determine the fraction of the total available climatic niche occupied by *Aeonium* on the Canary Islands, we first quantified the climatic space of the Canary Islands by selecting 10’000 spatially random localities across all islands (without considering where we sampled) and extracted the complete set of 20 climatic variables for each. We then extracted the major axes of variation by principal component analysis (PCA) and projected all localities to observe *Aeonium* distribution into the rotated PC space. For each PC axis, we computed the fraction of total climatic variation that is spanned by the sampling localities of the *Aeonium* species and computed the mean across axes, weighted by the variance explained by each axis. This analysis is conservative because it overestimates total climatic variation, as areas devoid of plant life (e.g. extremely cold or dry) are included. At the same time, it underestimates the climatic breadth of the genus, as it only comprises sampled presences, which is necessarily narrower than the total climatic breadth of the genus.

To test for phenotype–environment associations for univariate growth forms, we performed a phylogenetic PCA rotation of the climatic variables using taxon means that were scaled and centered (Revell, 2009). Among and within-species variations were estimated for all traits and climatic variables (**Supplementary Figures 1-31**). PC1 and PC2 scores were then used as input for a phylomorphospace analysis (*phytools* package) (Revell, 2012). In these plots, points indicate species’ phenotypes and lines reveal trajectories of evolution (Revell, 2014). Then, we performed the same phylomorphospace analysis as described above, using the 11 morphological traits as input for the phylogenetic PCA of morphology. Quantitative morphological traits were natural log-transformed to allow for comparing variation across traits measured on different scales (Lewontin, 1966). If growth forms represent discrete states in the morphospace, this analysis would reveal how morphological traits cluster the three growth forms. We tested climatic and morphological differences among growth forms with analyses of variance (ANOVA). Finally, we fitted phylogenetic generalized least squares (PGLS) regressions using the R package *caper* (Orme et al., 2018) of PC scores as a function of growth form for the first two PC axes of climate and morphology to test for phenotype-environment associations.

To get as close to trait utility in particular environments as possible, we tested correlations between individual climate and morphological variables. We used the PC axes of climate and morphological phylogenetic PCAs that showed statistically significant correlations and tested these against the three most explanatory variables of each axis. This allows a better understanding of what is behind the phenotype–environment correlations found before. Furthermore, we also performed the same (PGLS) analysis for each climatic variable against each morphological trait for a comprehensive overview (see **Supplementary Figures 32-42**). Significant interactions demonstrate that the morphological variable is associated differently with a climatic variable depending on growth form, which would suggest that the adaptive landscape of a trait depends on its whole plant context, i.e., what growth form.

## Results

### Climatic characterization and occupancy

The West and North-west sectors of the Canary Islands have a significant Atlantic influence, provided by the tradewind currents (**Figure 2A**). The first two principal components (PC) axes of all climatic variables capture 86.46% of the macroclimatic space variance (**Figure 2B**; **Supplementary Table 4**). The main drivers of PC1 are temperature ranges (BIO4 and BIO7) and temperatures of the coldest periods (BIO11 and BIO6). PC2 reflects North-South orientation, as its main drivers are solar radiation, precipitation seasonality (BIO15), and temperatures of the wettest and driest quarters (BIO08 and BIO09). Randomly drawn terrestrial points across the archipelago reveal a triangular shape (**Figure 2B**) with corners representing: (1) arid conditions (< 100 mm of precipitation per year, high PC1 scores), (2) warm and humid conditions (> 600 mm of precipitation per year, high PC2 scores), and (3) cold conditions with high thermal ranges, corresponding to high elevations (low PC1 and low PC2 scores).

*Aeonium* species have radiated into almost all potentially available climatic space (88% overall, **Figure 2B**). The available climatic space without *Aeonium* presence is either extremely dry (e.g., parts of the eastern islands) or alpine (e.g., the Teide volcano that reaches 3715 m a.s.l.). Both these environments are naturally very sparsely vegetated, suggesting the limits of the genus’ realized niche approaches the limits of plant life on the Canary Islands. In geographic space, the relatively small northern sectors of western islands with relatively high precipitation harbor the highest density and species richness of *Aeonium* (**Figure 2A**).

### Macroclimate of species and growth forms

**Figure 3:**
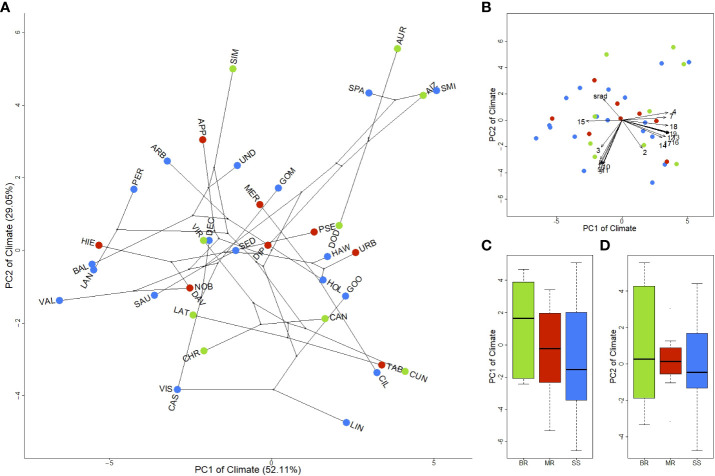
Phylogenetic principal component analysis of the climatic space of the Canarian *Aeonium*. **(A)** Phylogenetic principal component analysis of the climatic space of Canarian *Aeonium*. Each dot represents the mean of one taxon (species or subspecies). Lines indicate phylogenetic relationships among species. Taxa are indicated on each dot by a three-letter acronym (see **Supplementary Table 1**); **(B)** Loadings of the climatic variables of the phylogenetic principal component analysis. Numbers (1-19) indicate BIOs, and *srad* indicates the annual potential solar radiation (see **Supplementary Table 3**); **(C)** Distribution of growth forms along the first axis of the climatic space (PC1); **(D)** Distribution of growth forms along the second axis of the climatic space (PC2). Colors represent growth forms: green – BR (branching rosettes), red – MR (monocarpic rosettes), blue – SS (Shrubs or subshrubs). PCA loadings in **Supplementary Table 5**.

To quantify phenotype–environment associations, we first considered the dominant axes of climatic differentiation among species and then determined whether an overall association with growth forms exists. The first two phylogenetic PCA axes of taxon-mean climatic data well-approximates diversity among species, as they capture 81.16% of the variation (**Figure 3** and **Supplementary Table 5**). Species are widely dispersed throughout climatic space as opposed to forming clusters. PC1 is mostly a precipitation gradient (e.g., BIO19, BIO16, BIO12). Low PC1 scores represent low precipitation seasonality (BIO15), associated with high isothermality (BIO03) and relatively high temperatures, especially during the coldest periods (e.g., BIO06, BIO11; **Figure 3B**). On the other hand, PC2 represents a temperature gradient and solar radiation. High temperatures correspond to low PC2 values, whereas high solar radiation represents the positive end of the PC2 axis. As a result, the negative end of PC1 represents markedly dry environments occupied by species adapted to very xerophytic environments, such as *A. valverdense*, *A. lancerottense*, *A. balsamiferum* or *A. hierrense*, endemic to the driest islands: Lanzarote, Fuerteventura and El Hierro (**Figures 2A**, **3A**). High PC1 scores and low PC2 scores indicate high precipitation combined with high temperatures (e.g., BIO05 or BIO10 in PC2), typical of sub-tropical environments, with species associated with the wet laurel forest (e.g., *A. lindleyi* ssp. *lindleyi*, *A. ciliatum*, *A. cuneatum* and *A. tabuliforme*; **Figures 3A, B**). High PC1 scores combined with high PC2 scores represent high precipitation and strong temperature seasonality (BIO04), with typical mid-elevation Canarian pine forest species (*A. aureum*, *A. spathulatum*, *A. aizoon*, and *A. smithii*).

Growth forms do not form three discrete clusters; instead, they are dispersed throughout the climatic space, indicating no simple overarching structuring effect of growth form on climatic niche evolution (**Figures 3A, C, D**). Furthermore, we found no significant differences in mean PC scores among the three growth forms (**Figures 3C, D**; **Table 1**). Congruently, relationships among species (illustrated by lines representing phylogenetic relationships in **Figure 3A**) indicate that climatic niche evolution has occurred frequently and in multiple directions. Moreover, species-pairs with disparate growth forms may occur in similar climates, e.g., *A. aizoon* and A. smithii (BR and SS), *A. pseudourbicum* and *A. dodrantale* (MR and BR), *A. ciliatum* and *A. tabuliforme* (SS and MR, both climatically close to *A. cuneatum*, a BR species; see **Figure 3A**).

**Table 1:** Results of ANOVAs and Tukey HSD *post-hoc* testing for PC1 and PC2 axes of climate and morphology among growth forms.

Variables	Df	F	*p*-value	Growth forms		Difference	*p*-value	95% confidence interval	
								Lower	Upper
PC1 Climate	2, 34	1.297	0.287	BR	MR	-1.350	0.656	-5.108	2.408
				BR	SS	-2.039	0.256	-5.143	1.066
				MR	SS	-0.689	0.861	-3.925	2.546
PC2 Climate	2, 34	0.386	0.683	BR	MR	-0.565	0.901	-3.745	2.616
				BR	SS	-0.938	0.659	-3.566	1.689
				MR	SS	-0.374	0.940	-3.112	2.364
PC1 Morphology	2, 34	22.41	<0.001 ***	BR	MR	2.304	0.039 *	0.100	4.508
				BR	SS	-2.692	0.003 **	-4.512	-0.872
				MR	SS	-4.996	< 0.001 ***	-6.893	-3.099
PC2 Morphology	2, 34	2.695	0.082	BR	MR	-0.176	0.979	-2.355	2.004
				BR	SS	-1.480	0.124	-3.280	0.321
				MR	SS	-1.304	0.219	-3.180	0.572

Stars indicate significance level (*, p < 0.05; **, p < 0.01; ***, p < 0.001).

### Morphological profile of growth forms

**Figure 4:**
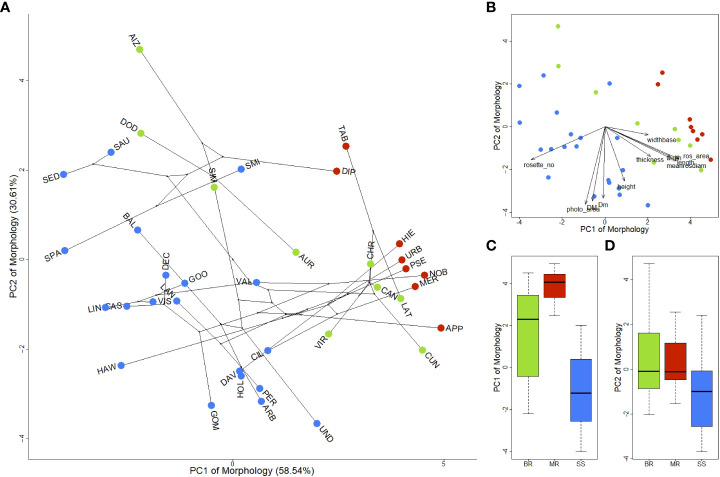
Phylogenetic principal component analysis of the morphological space of the Canarian *Aeonium*. **(A)** Phylogenetic principal component analysis of the morphological space of Canarian *Aeonium*. Each dot represents the mean of one taxon (species or subspecies). Lines indicate phylogenetic relationships among species. Taxa are indicated on each dot by a three-letter acronym (see **Supplementary Table 1**); **(B)** Loadings of the morphological traits of the phylogenetic principal component analysis (see **Supplementary Table 2** for traits’ abbreviations); **(C)** Distribution of growth forms along the first axis of the morphological space (PC1); **(D)** Distribution of growth forms along the second axis of the morphological space (PC2). Colors represent growth forms: green – BR (branching rosettes), red – MR (monocarpic rosettes), blue – SS (Shrubs or subshrubs). PCA loadings in **Supplementary Table 6**.

The growth form classification used in this study is specific for *Aeonium* (Lems, 1960) and used the primary growth forms observed, avoiding sub-categorizations. The classification is based on the branching pattern: unbranched (MR), stoloniferous plants that branch at or below the surface (BR) and stemmed plants that branch above the surface (SS). The analysis of the phylomorphospace shows that individual morphological traits explain growth form diversity, specifically the first axis of the PCA, which captures alone 58.54% of the morphological variation, mainly through a trade-off between rosette size and number (**Figures 4A, B** and **Supplementary Table 6**). High PC1 scores are explained by high rosette diameter, rosette area and leaf length; low PC1 scores are explained by rosette number (**Figure 4B**; **Supplementary Table 6**). Hence, highly branched species (namely SS) form a cluster towards the negative end of PC1, whereas the opposing end includes all single-rosetted species (MR). On the other hand, low PC2 scores capture the size of the plant, specifically by high photosynthetic area, plant diameter (both maximum and minimum) and plant height (**Figure 4B**; **Supplementary Table 6**). The negative end of PC2 thus includes the largest species, mostly large SS (e.g., *A. undulatum*, *A. arboreum*, *A. gomerense*). In contrast, the positive end includes small-stature taxa, mostly BR (e.g., *A. aizoon*, *A. dodrantale* and *A. simsii*), stemless MR (*A. tabuliforme* and *A. diplocyclum*) and dwarf SS (e.g., *A. smithii*, *A. saundersii*, *A. sedifolium*). This result demonstrates that growth forms represent arrays of morphological traits with clear delimitations among the three growth forms regarding the rosette trade-off (**Figure 4C**). We found statistically significant differences in PC1 of morphology according to growth form (*p* < 0.001; **Table 1**). Furthermore, we found significant pairwise differences among all growth forms, with SS and MR being the most dissimilar (*p* < 0.001), followed by SS and BR (*p* = 0.003), and finally, MR and BR (*p* = 0.039; **Table 1**). Lower statistically significant differences between MR and BR could be explained by the rosette trade-off: BR species tend to have the largest rosettes (except dwarf species like *A. aizoon*, *A. dodrantale* or *A. simsii*), thus producing a much smaller number of rosettes. The PC2 of morphology, on the other hand, does not show significant differences (*p* = 0.082), including no pairwise differences among growth forms (**Table 1**).

### Evidence of adaptations to climate

**Figure 5:**
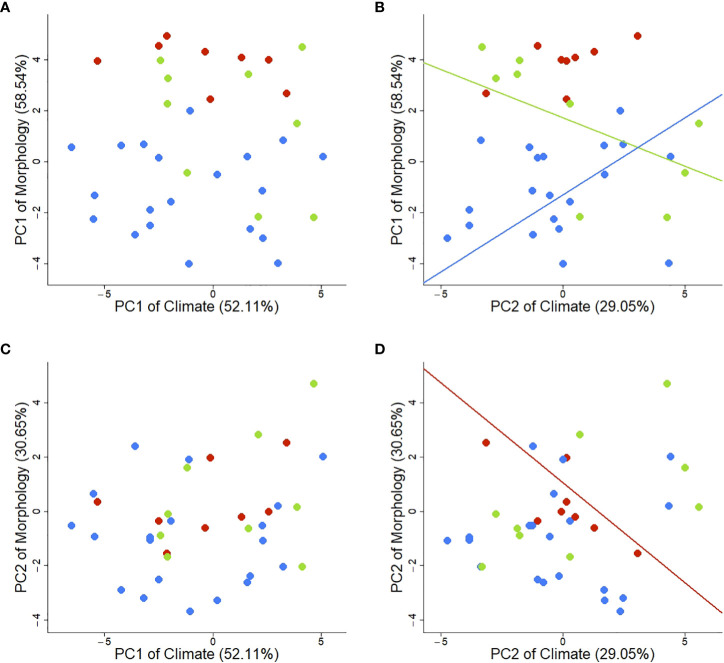
Interactions and PGLS regressions between first and second components of climatic and morphological spaces. **(A)** PC1 of Climate and PC1 of Morphology. **(B)** PC2 of Climate and PC1 of Morphology (BR: Intercept = 1.727, *p* = 0.025; Slope = -0.380, *p* = 0.037; SS: Intercept = -1.302, *p* < 0.001, Slope = 0.604, *p* = 0.009). **(C)** PC1 of Climate and PC2 of Morphology. **(D)** PC2 of Climate and PC2 of Morphology (MR: Intercept = 1.057, *p* = 0.404; Slope = -0.735, *p* = 0.029). Lines represent statistically significant regressions (*p* < 0.05). Colors indicate different growth forms: green – BR (branching rosettes), red – MR (monocarpic rosettes), blue – SS (shrubs or subshrubs).

We compared the PC1 and PC2 axes of both climate and morphology and found statistically significant correlations between climate (PC2) and individual morphological traits (PC1) in two growth forms (**Figure 5B**). As explained above, the PC2 of climate represents a temperature and solar radiation gradient with high temperatures on the negative end and high solar radiation on the positive end. The PC1 of morphological traits represents the trade-off between rosette number and rosette size. Though no correlation was found when using all taxa irrespective of their growth form (no overall correlation between climate and morphology), we find statistically significant relations when growth forms are considered in the model (**Table 2**). Specifically, we found a positive association for SS (*p* = 0.009) and a negative association for BR (*p* = 0.025; **Figure 5B**; **Table 2**). Contrastingly, MR show a marginally significant signal (*p* = 0.056; **Table 2**) and is the only growth form to have a statistically significant correlation between PC2 of climate and PC2 of morphology (*p* = 0.029; **Figure 5D**; **Table 2**).

**Table 2:** Phylogenetic generalized least squares models (PGLS) results of climate and morphology interactions among growth forms.

Response variables	Predictors	Estimate	Std. Error	*t-*value	*p-*value	Residual std. error	Multiple R^2^	Adjusted R^2^	F-statistic	*p*-value
PC1 Morphology	PC1 Climate x BR	1.086	0.914	1.188	0.244	1.917	0.5889	0.5226	8.88	< 0.001 ***
	PC1 Climate x MR	-0.188	0.308	-0.610	0.547					
	PC1 Climate x SS	0.047	0.234	0.201	0.842					
	PC2 Climate x BR	1.727	0.732	2.360	0.025*	1.633	0.6809	0.6294	13.23	< 0.001 ***
	PC2 Climate x MR	0.698	0.351	1.986	0.056					
	PC2 Climate x SS	0.604	0.217	2.781	0.009**					
PC2 Morphology	PC1 Climate x BR	0.638	0.888	0.719	0.478	1.786	0.2549	0.1347	2.12	0.089
	PC1 Climate x MR	0.210	0.272	0.773	0.445					
	PC1 Climate x SS	-0.081	0.206	-0.393	0.697					
	PC2 Climate x BR	0.381	0.830	0.459	0.650	1.644	0.3505	0.2457	3.346	0.016*
	PC2 Climate x MR	-0.735	0.321	-2.292	0.029*					
	PC2 Climate x SS	-0.330	0.208	-1.586	0.123					

Stars indicate significance level (*, p < 0.05; **, p < 0.01; ***, p < 0.001).

Further bivariate correlations between the climatic variables and morphological traits corroborate the pattern observed in **Figure 5B**: morphological traits of BR and SS (namely rosette size and number) exhibit opposite relationships with climate (**Supplementary Figures 32-42**). In contrast, the interaction of MR with the climate acts on a different morphological level: plant size (represented by the PC2 of morphology). This analysis shows how traits interact differently with the climate according to their growth form. Peculiarly, rosette number demonstrates more plasticity towards temperature changes than rosette size (diameter and area). More specifically, SS have more and smaller rosettes with high temperatures. On the other hand, BR have fewer but bigger rosettes in similar environments, demonstrating opposite phenotype-environment associations (**Figure 5B**). More interestingly, MR interact with the same environment on a different level by the overall plant size with increasing solar radiation.

The relation between solar radiation and the rosette size-number trade-off (PC1 morphology) shows, once more, contrasting behaviors among growth forms. Branching rosettes showed more but smaller rosettes when occupying environments with higher solar radiation (mid-elevation species); in similar contexts, SS showed fewer but bigger rosettes (**Figure 5B**, but see also Supplementary Figures 32, 36, and 41). Similarly to SS, MR evidenced increasing plant size by increasing plant height and rosette area (**Figure 5D** but see also **Supplementary Figures 33-42**). Even though MR are characterized by having a single rosette (which could explain the lack of response since rosette number is the major driving factor of PC1 of morphology), with increasing solar radiation, the plants were larger.

## Discussion

Species diversification through adaptation to the local environment is a central premise of adaptive radiations (Grant, 1981; Schluter, 2000; Losos and Ricklefs, 2009). Growth forms have long been used to categorize morphological divergence in plants and are frequently recognized as adaptations to environments (Lems, 1960; Nürk et al., 2019; Schenk, 2021). In this study, we demonstrate that the intimate relationship that growth forms share with the environment is not a direct result of adaptation per se. Instead, the identified climatic dimensions do not affect the distribution of growth forms. However, growth forms modulate the response of specific morphologic traits to the local climate by developing growth form-specific trait adaptations to similar climatic conditions. Thus, our results indicate that growth forms have an indirect rather than direct association with climate. The phenotypic adaptations to the environment occur in individual traits and are, in this context, growth form dependent. We also found that multi-rosetted growth forms (i.e., BR and SS), showed, without exception, opposing responses to the environmental conditions. Furthermore, single-rosetted species (MR) interact with the same climate on a different morphological axis. Consequently, the response of growth forms to the environment depends on their intrinsic morphology and how traits interact with each other.

*Aeonium* is widely distributed across all islands along a macroclimate gradient of precipitation, representing the primary axis of niche divergence among species (**Figure 3**). The second dimension of the climatic distribution of *Aeonium* is defined by the local climate, as it represents the local climatic variation explained essentially by topographic complexity (e.g., reflected in solar radiation) (Otto et al., 2016; Testolin et al., 2021). Our results show that specific morphological traits interact with the local climate, playing an essential role in trait adaptation. These findings are consistent with previous studies, which have shown that local climate is a significant driver of trait diversification and, thus, of speciation and adaptation (Jorgensen, 2002; Bruelheide et al., 2018; Testolin et al., 2021). At the same time, the macroclimate delimitates species’ climatic preferences and is thus responsible for the overall distribution pattern of species (Zimmermann et al., 2009; Miguez-Macho and Fan, 2021).

Morphological diversity is structured in the three growth forms based on the rosette number and size trade-off (**Figure 4**). Rosette size (specifically rosette diameter and rosette area) forms a strong, negative association with rosette number because individual shoot apical meristem size trades off with meristem number through the number of branching events (Whitman and Aarssen, 2010). These two traits alone segregate most species in different growth forms (**Figure 4**). This trade-off is the central axis of the interaction of traits with the microclimate and represents the axis of phenotypic adaptation to the environment. Specifically, we found that in BR, rosette size increases in environments with high temperatures and low solar radiation (i.e., north-facing flanks of the islands; **Figure 5B**); thus, rosette number decreases.

On the other hand, we found that SS in the same conditions (high temperatures and low solar radiation) increased rosette number while reducing rosette size. The biological limitation of MR species, having by definition one single rosette, implies minimal variation on the rosette number-size trade-off. Nevertheless, MR species interact with the microclimate through a different morphological aspect, i.e., plant size. This particular adaptation is in line with their primary ecological strategy, as monocarpic plants prioritize dispersal and fast colonization (Mudrák et al., 2021).

Monocarpic species (MR) rely on a lifetime investment strategy that results in a single “big bang” reproduction event and subsequent senescence – a remarkably successful strategy toward populating unstable and highly disturbed environments given the fast development innate to this growth form (Jorgensen and Olesen, 2001; Kakishima et al., 2019; Anest et al., 2021). Furthermore, monocarpic plants are prevalent in evolutionary radiations in island-like environments, given their great capacity for rapidly colonizing new territory (particularly new islands) (Givnish, 2010; Kakishima et al., 2019). On the other end of the spectrum, multi-rosetted growth forms (BR and SS) produce a comparatively low number of reproductive units per season (Lavorel et al., 1997; Mudrák et al., 2021), investing in establishing populations with higher longevity and thus associated with low-disturbance environments, where competitive performance is more important than dispersal (Mudrák et al., 2021). Adapting to local conditions becomes essential for settler species in this complex ecological context. On the other hand, fast colonizer species prioritize the investment in reproduction and seed dispersal traits, like plant height (Mudrák et al., 2021). The interactions between plant height and climatic variables among monocarpic species suggest that specific population dynamics strategies could intrinsically relate to distinct ecological strategies (Rudolf and Rasmussen, 2013). While MR are fast colonizers through massive seed production and dispersal, BR and SS share a “settler” strategy that prioritizes growth over reproduction through constant branching while having contrasting responses to analogous environments. This clear relation of contrasting trait adaptations inflected by growth forms could suggest latent ecological strategies (Lavorel et al., 1997; van der Plas, 2019). Considering plant traits are adaptations to abiotic factors (Caruso et al., 2020), growth forms could also play a role in adapting to complex biotic interactions, namely interspecific competition avoidance. In this scenario, competition avoidance is attained through contrastive trait responses to the same environment, a commonly recognized indication of complementary ecosystem strategies (Weber et al., 2017; Ronco et al., 2021). While competition avoidance was not tested in this study, it is an ecological strategy that allows spatial co-occurrence within species of the same lineage through divergent resource use, ultimately triggering diversification (Wilson, 1961; Herrmann et al., 2021). Although competition avoidance is not a novel idea in island radiation contexts (Herrmann et al., 2021), it is a largely understudied topic that could disclose how phylogenetically close species co-occur without actively competing while corroborating the known fact that plant radiations certainly have high growth form diversity.

In summary, growth form divergence has a strikingly multifaceted, subtle, but critical role in plant radiations. This study demonstrates that growth forms dictate the direction of trait adaptations to microclimate rather than being adaptations per se. The most striking example is the opposite direction of adaptation for the same traits in different growth forms with similar ecological strategies (“settler” species, in this case, BR versus SS). In analogous climatic conditions, these growth forms respond through opposing directions of the rosette number-size trade-off. At the same time, MR interacts with the climate through plant size. The architectural limitations of each growth form determine trait interactions and trade-offs responsible for opposing environmental responses (**Figure 5**). This unforeseen result indicates that the whole (growth form) is different from the sum of its parts (traits) and demonstrates that the response of traits to the local climate (determined by the growth form) originates novel morphological solutions as adaptations to similar environmental conditions. Growth form shifts represent a change of ecological dimension perceived by the plants. Such shifts open new adaptive landscapes, allowing not only competition avoidance and sympatric evolution, but ultimately acting as a diversification trigger.

## Data availability statement

The raw data supporting the conclusions of this article will be made available by the authors, without undue reservation.

## Author contributions

PS and JV conceived and designed the project. PS, MB, TM, and HS gathered data under the supervision of GK, CB, and JV. PS and JV designed and executed the statistical analyses. PS created the tables and figures. PS wrote the first version of the manuscript with the input of JV and CB. All authors contributed to and approved the final version of the manuscript.

## Funding

This work was funded by Fundação para a Ciência e a Tecnologia, I.P./MCTES through National Funds UIDB/00329/2020). PS was supported by Fundação para a Ciência e Tecnologia (FCT) with the PhD grant no. PD/BD/128367/2017. Fieldwork was funded by the Stiftung zur Förderung der Pflanzenkenntnis, the Centre for Ecology, Evolution and Environmental Changes (cE3c unit funding by FCT, UID/BIA/00329/2019 and UID/BIA/00329/2013), and the University of Basel. JV was supported in part by Swiss National Science Foundation grant 310030_185251.

## Acknowledgments

The authors would like to address their gratitude to Ángel Bañares-Baudet and Alfredo Reyes-Betancort. The authors thank the Canarian authorities for the collection permits that allowed the conduction of this study, namely the Consejerías de Medio Ambiente from the Cabildo Insular of El Hierro, Gran Canaria, La Gomera, La Palma, Lanzarote and Tenerife.

## Conflict of interest

The authors declare that the research was conducted in the absence of any commercial or financial relationships that could be construed as a potential conflict of interest.

## Publisher’s note

All claims expressed in this article are solely those of the authors and do not necessarily represent those of their affiliated organizations, or those of the publisher, the editors and the reviewers. Any product that may be evaluated in this article, or claim that may be made by its manufacturer, is not guaranteed or endorsed by the publisher.
