# Supplementary material for: Plant growth forms dictate adaptations to the local climate
Source: Front Plant Sci. 2022 Nov 21;13:1023595. doi: 10.3389/fpls.2022.1023595 (PMC9720395; doi:10.3389/fpls.2022.1023595)
Supplement: Supplementary file 2 [file DataSheet_1.pdf]

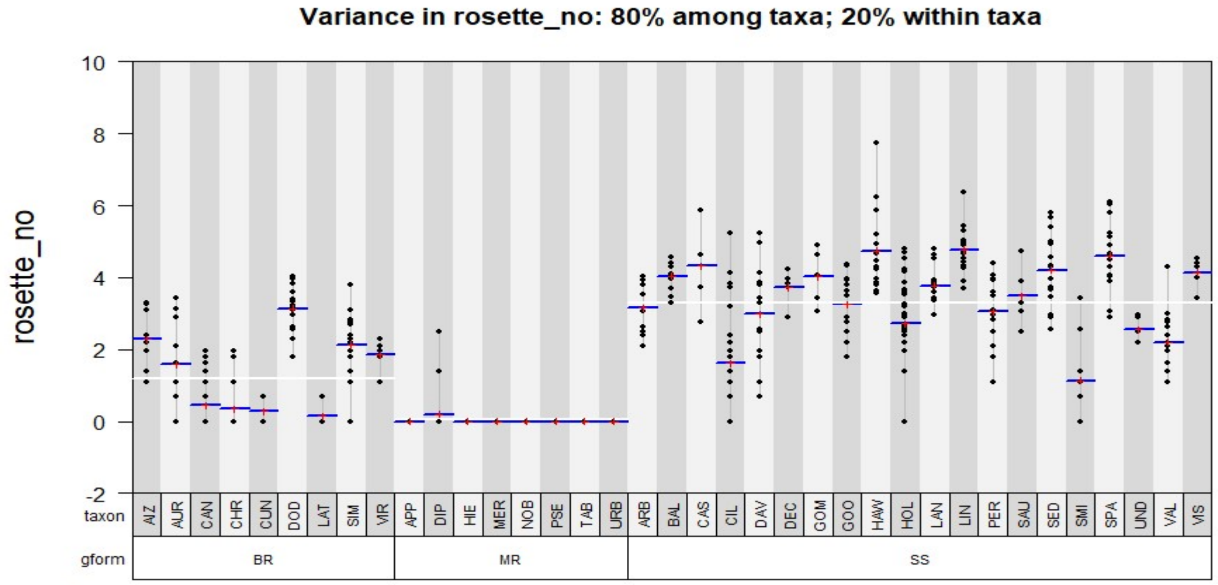

Supplementary Figure 1. Among and within taxa variance of rosette number. Rosette number values are log-transformed. Each point represents one individual; blue lines represent the taxon-mean values; white lines represent the growth form mean values.

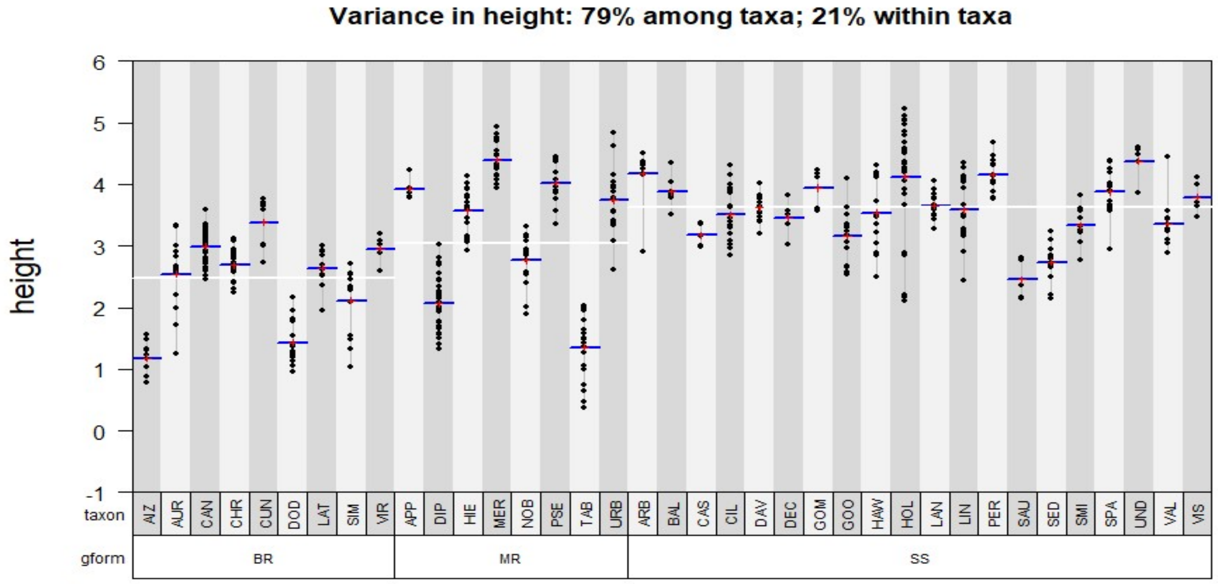

Supplementary Figure 2. Among and within taxa variance of plant height. Plant height values are log-transformed. Each point represents one individual; blue lines represent the taxon-mean values; white lines represent the growth form mean values.

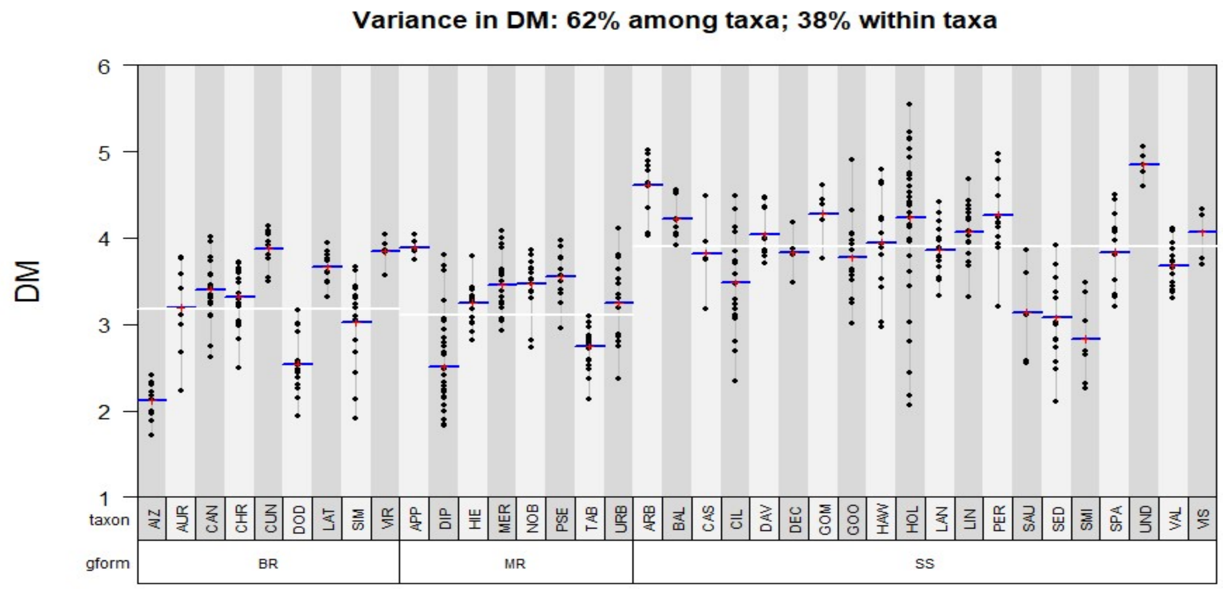

Supplementary Figure 3. Among and within taxa variance of maximum plant diameter. Maximum plant diameter values are log-transformed. Each point represents one individual; blue lines represent the taxon-mean values; white lines represent the growth form mean values.

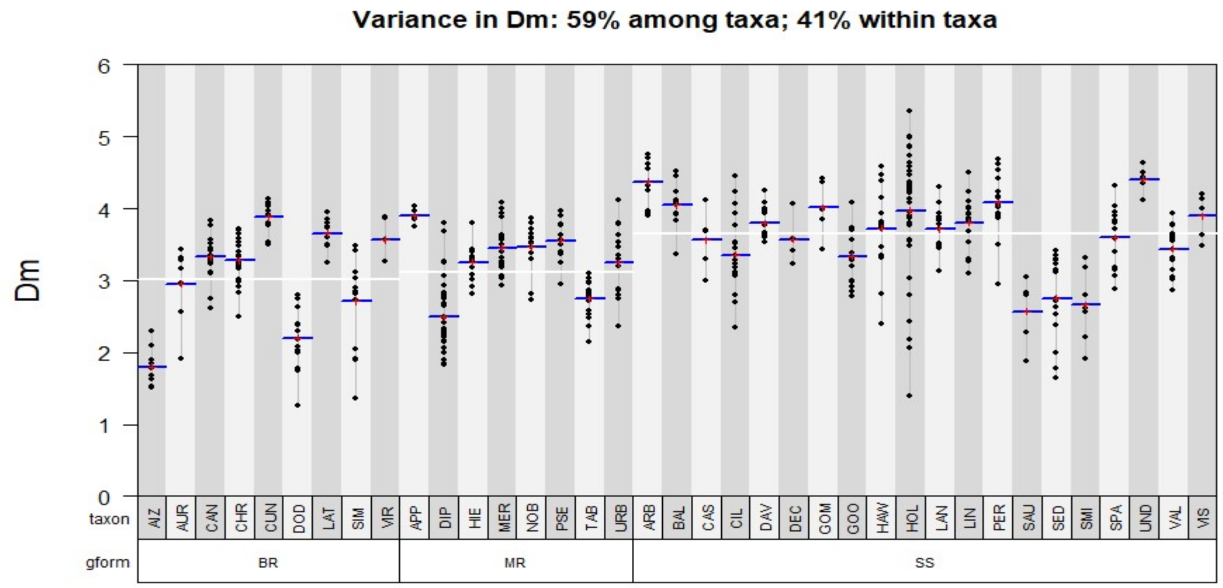

Supplementary Figure 4. Among and within taxa variance of minimum plant diameter. Minimum plant diameter values are log-transformed. Each point represents one individual; blue lines represent the taxon-mean values; white lines represent the growth form mean values.

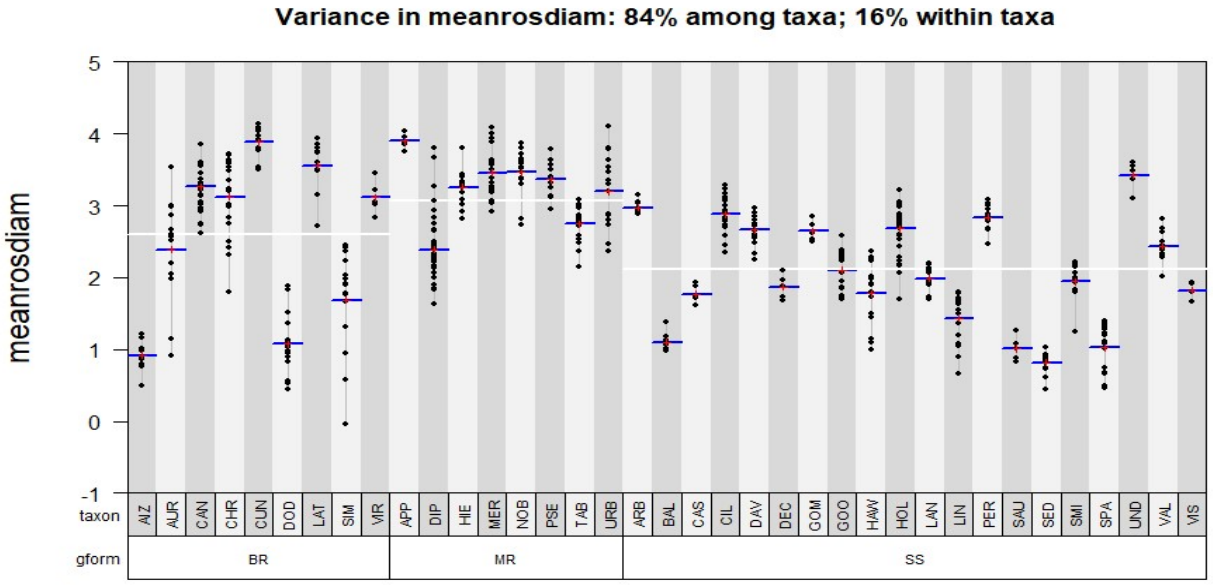

Supplementary Figure 5. Among and within taxa variance of rosette diameter. Rosette diameter values are log-transformed. Each point represents one individual; blue lines represent the taxon-mean values; white lines represent the growth form mean values.

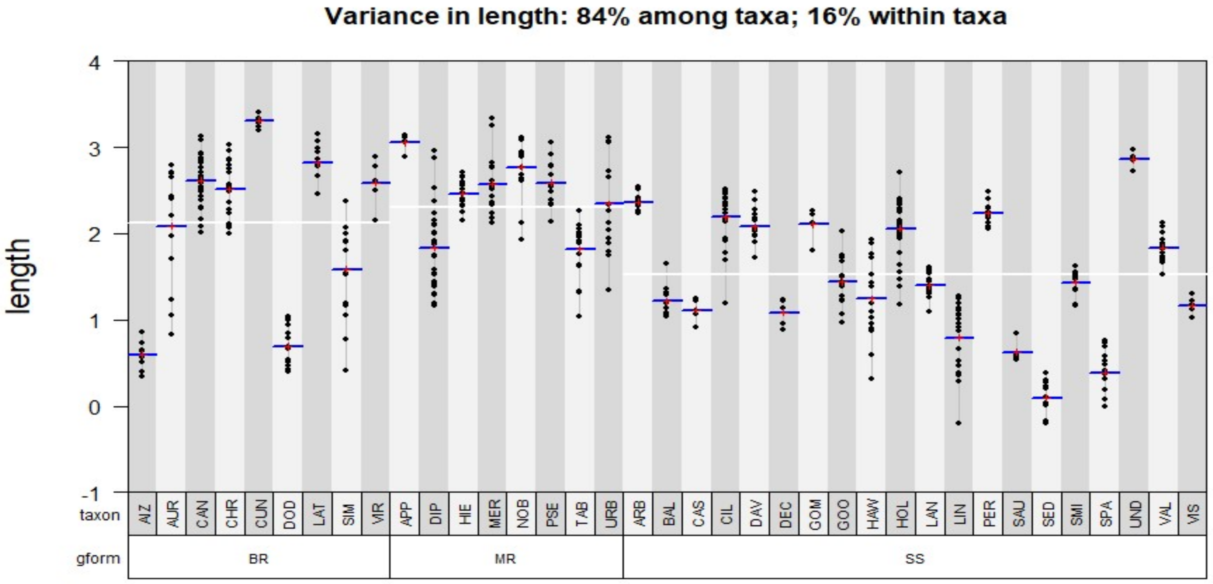

Supplementary Figure 6. Among and within taxa variance of leaf length. Leaf length values are log-transformed. Each point represents one individual; blue lines represent the taxon-mean values; white lines represent the growth form mean values.

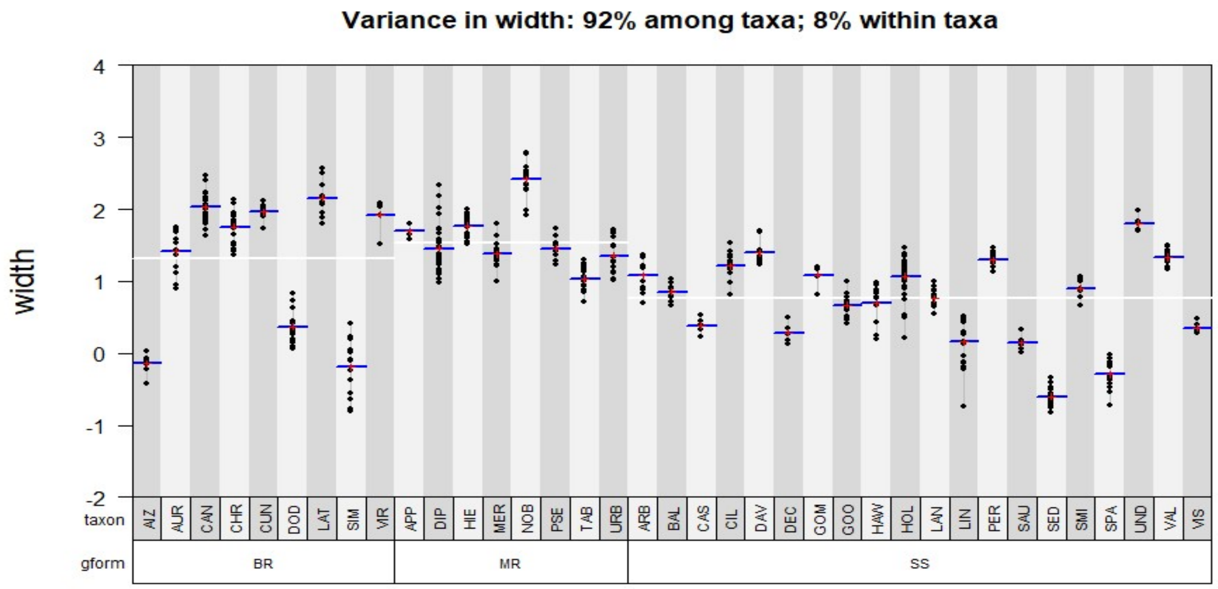

Supplementary Figure 7. Among and within taxa variance of leaf width. Leaf width values are log-transformed. Each point represents one individual; blue lines represent the taxon-mean values; white lines represent the growth form mean values.

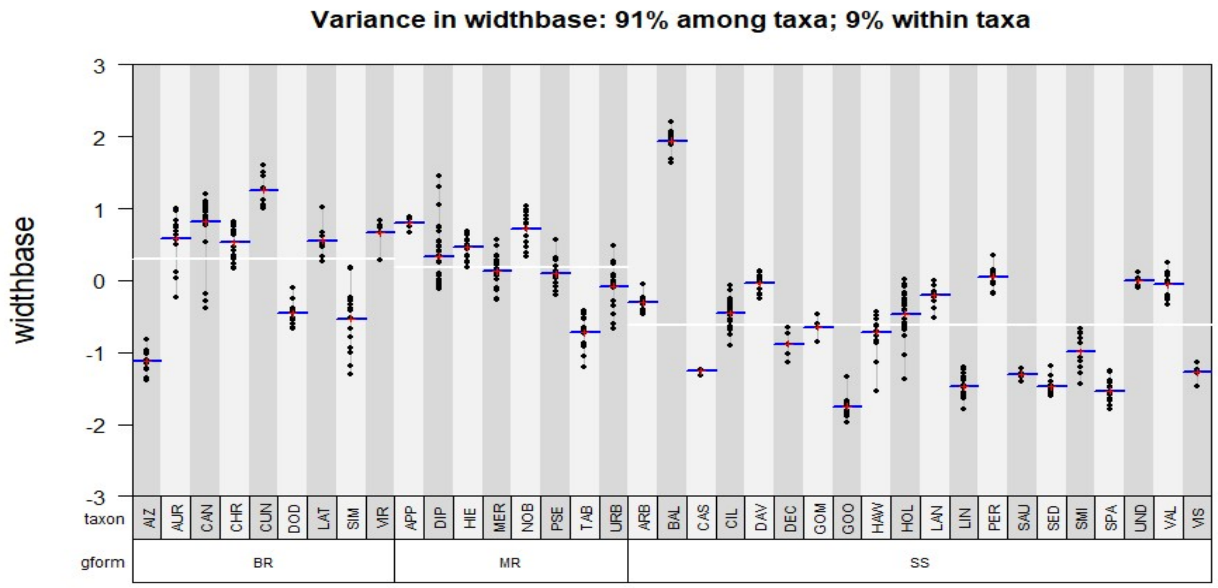

Supplementary Figure 8. Among and within taxa variance of petiole width. Petiole width values are log-transformed. Each point represents one individual; blue lines represent the taxon-mean values; white lines represent the growth form mean values.

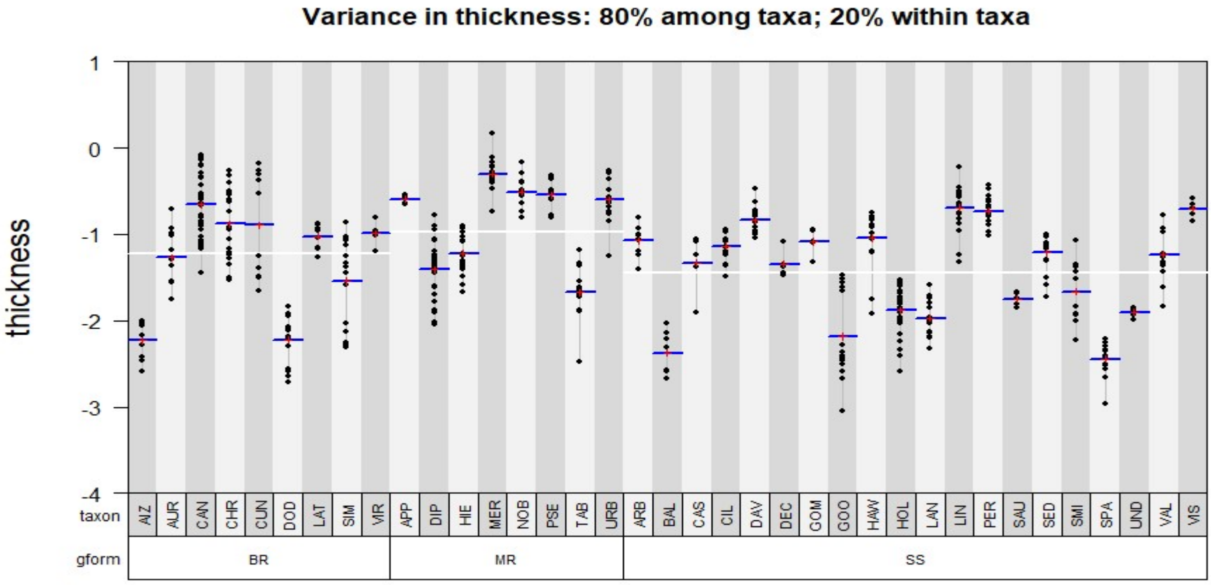

Supplementary Figure 9. Among and within taxa variance of leaf thickness. Leaf thickness values are log-transformed. Each point represents one individual; blue lines represent the taxon-mean values; white lines represent the growth form mean values.

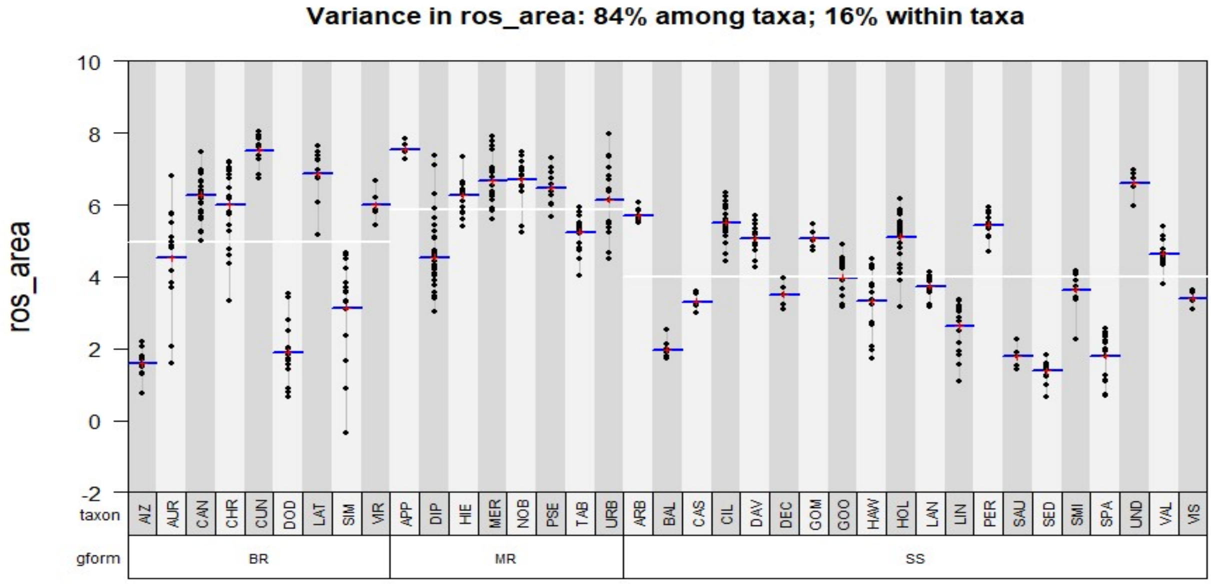

Supplementary Figure 10. Among and within taxa variance of rosette area. Rosette area values are log-transformed. Each point represents one individual; blue lines represent the taxon-mean values; white lines represent the growth form mean values.

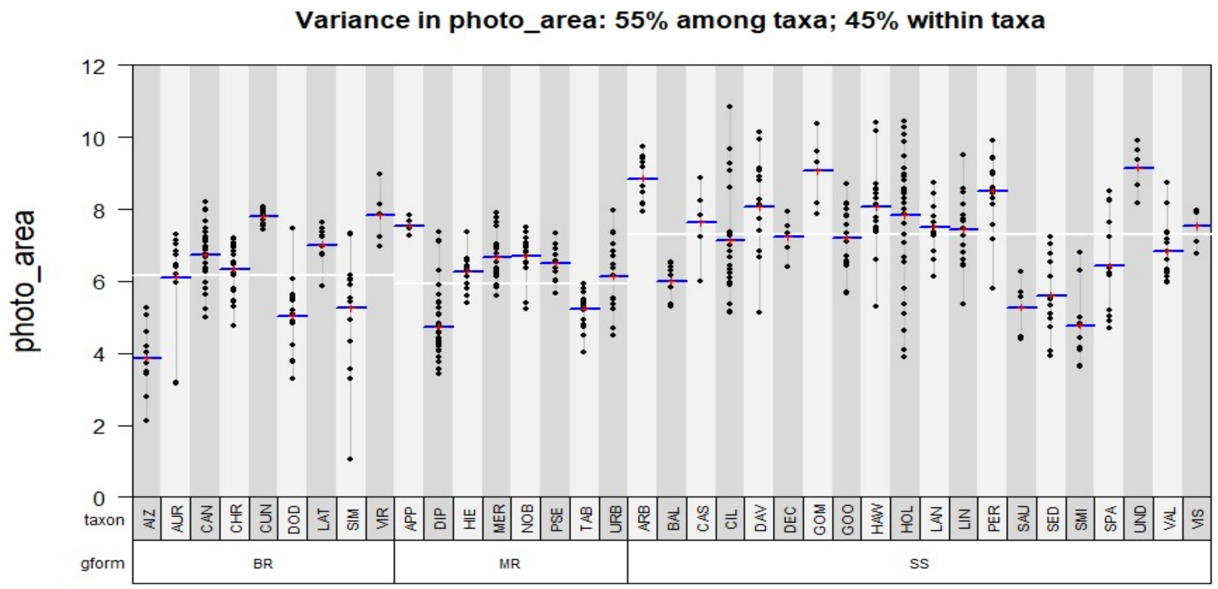

Supplementary Figure 11. Among and within taxa variance of photosynthetic area. Photosynthetic area values are log-transformed. Each point represents one individual; blue lines represent the taxon-mean values; white lines represent the growth form mean values.

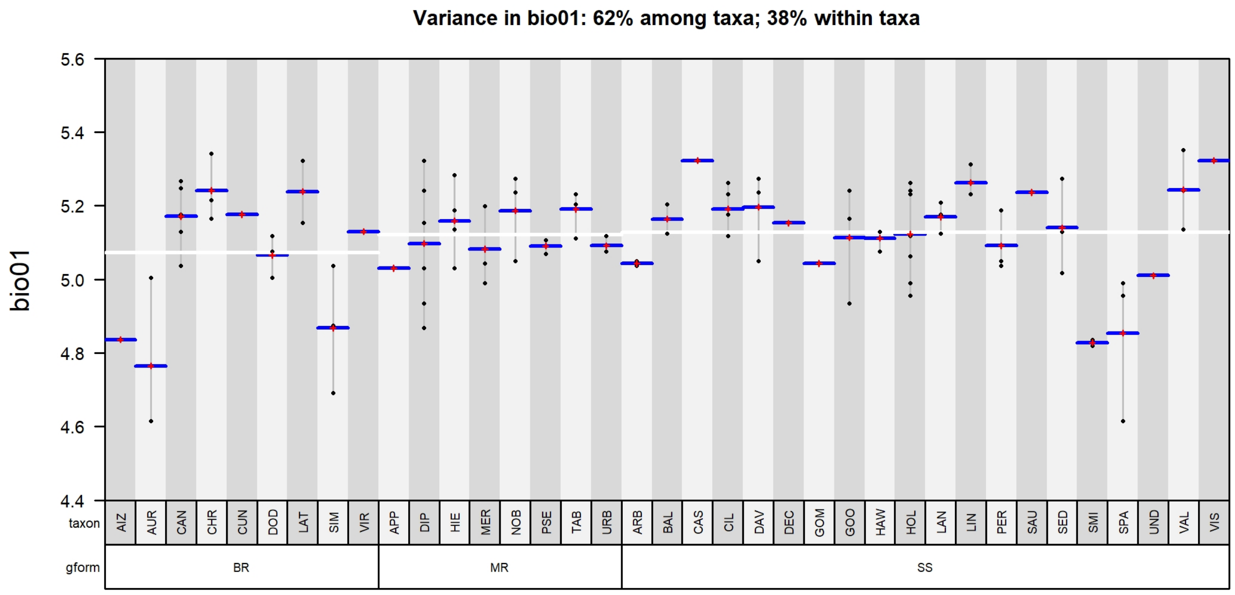

Supplementary Figure 12. Among and within taxa variance of BIO01 (annual mean temperature). BIO01 values are log-transformed. Each point represents one population; blue lines represent the taxon-mean values; white lines represent the growth form mean values.

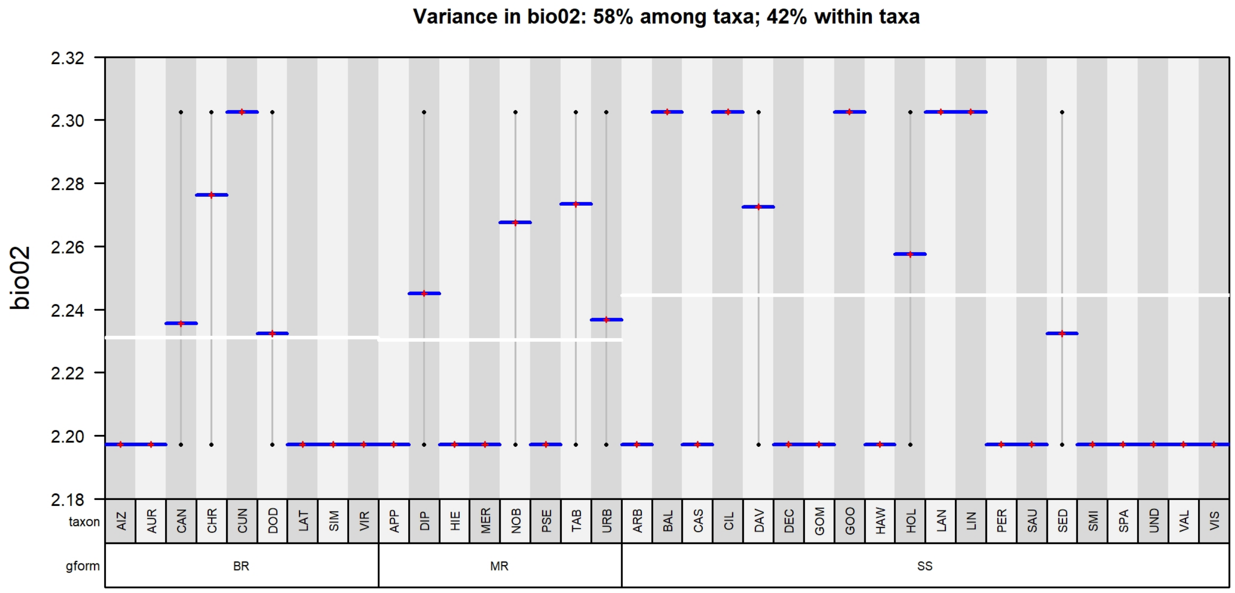

Supplementary Figure 13. Among and within taxa variance of BIO02 (mean diurnal range). BIO02 values are log-transformed. Each point represents one population; blue lines represent the taxon-mean values; white lines represent the growth form mean values.

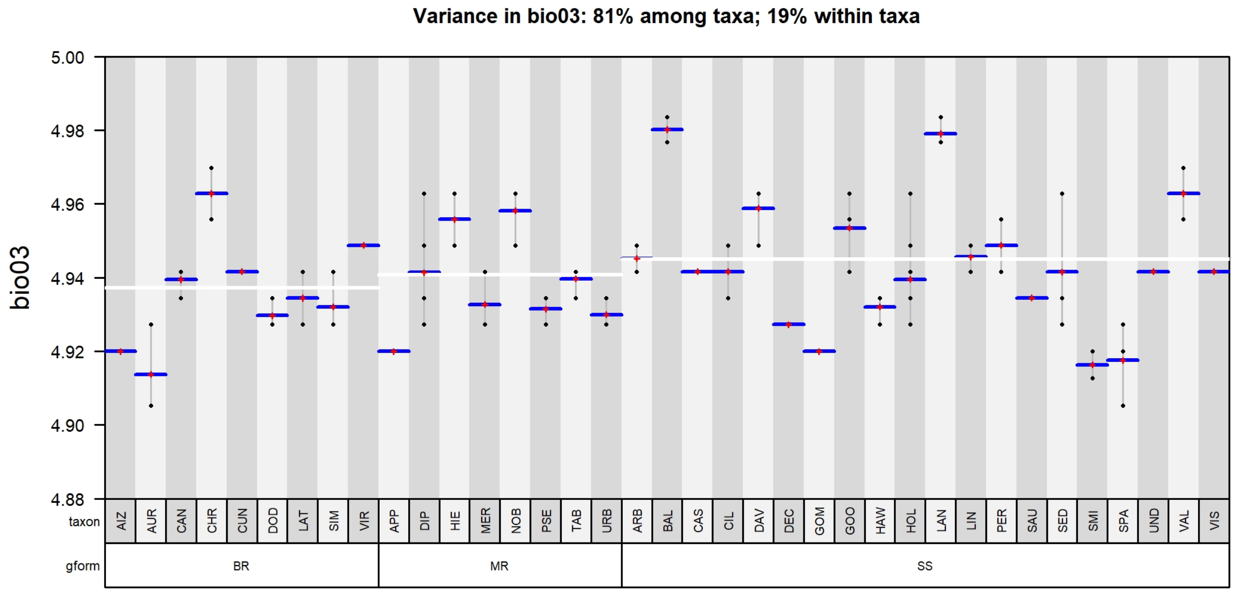

Supplementary Figure 14. Among and within taxa variance of BIO03 (isothermality). BIO03 values are log-transformed. Each point represents one population; blue lines represent the taxon-mean values; white lines represent the growth form mean values.

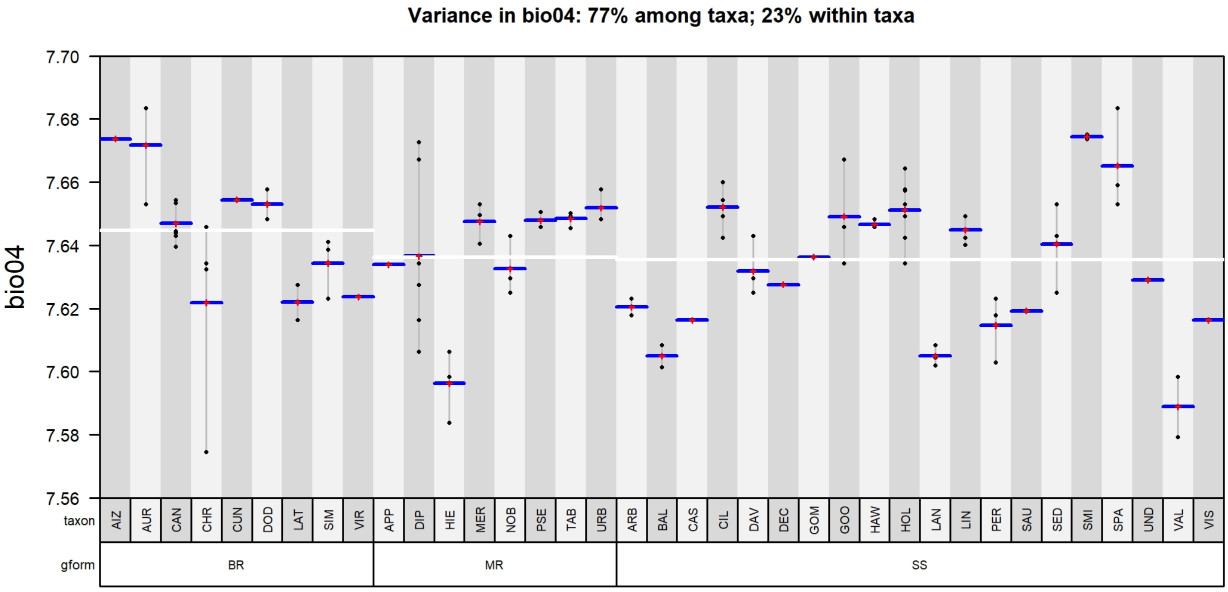

Supplementary Figure 15. Among and within taxa variance of BIO04 (temperature seasonality). BIO04 values are log-transformed. Each point represents one population; blue lines represent the taxon-mean values; white lines represent the growth form mean values.

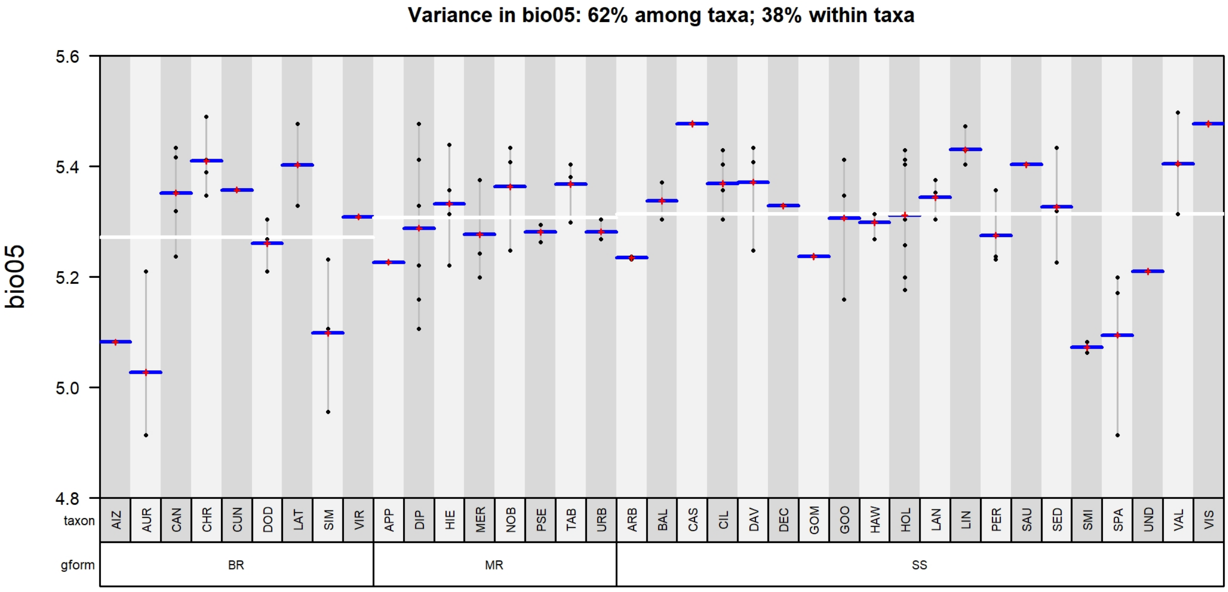

Supplementary Figure 16. Among and within taxa variance of BIO05 (maximum temperature of the warmest month). BIO05 values are log-transformed. Each point represents one population; blue lines represent the taxon-mean values; white lines represent the growth form mean values.

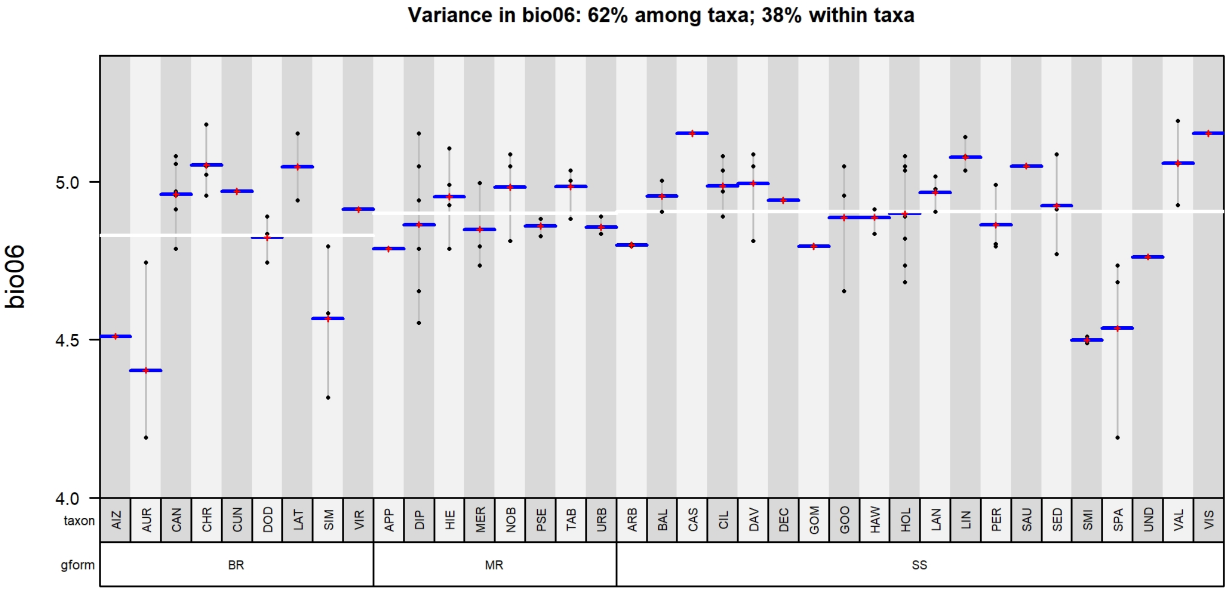

Supplementary Figure 17. Among and within taxa variance of BIO06 (minimum temperature of the coldest month). BIO06 values are log-transformed. Each point represents one population; blue lines represent the taxon-mean values; white lines represent the growth form mean values.

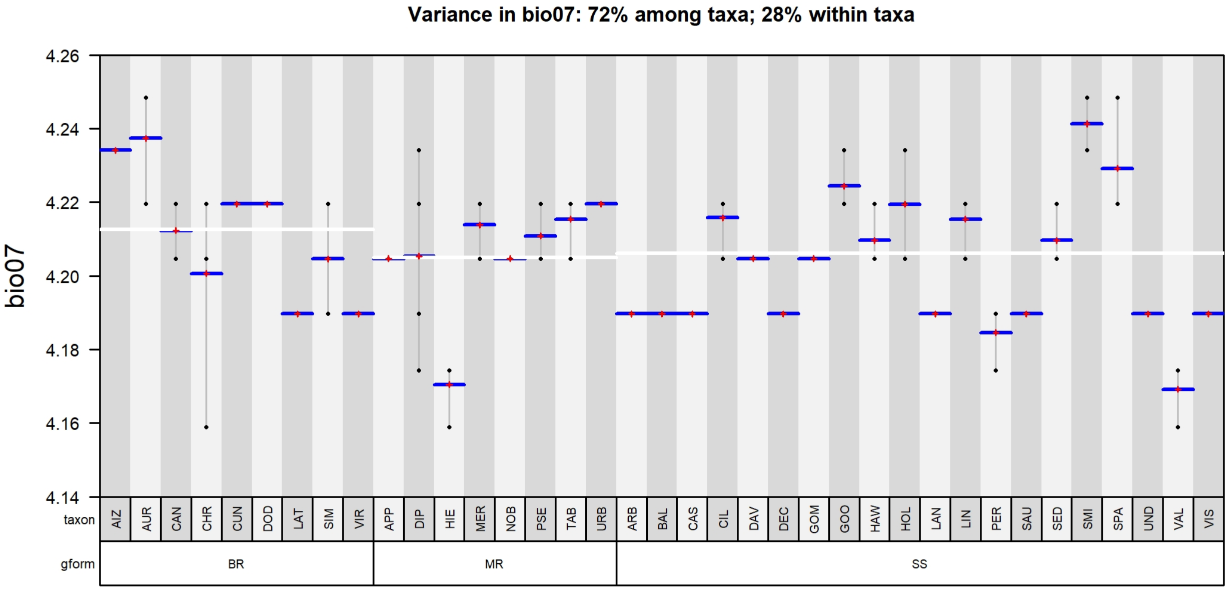

Supplementary Figure 18. Among and within taxa variance of BIO07 (temperature annual range). BIO07 values are log-transformed. Each point represents one population; blue lines represent the taxon-mean values; white lines represent the growth form mean values.

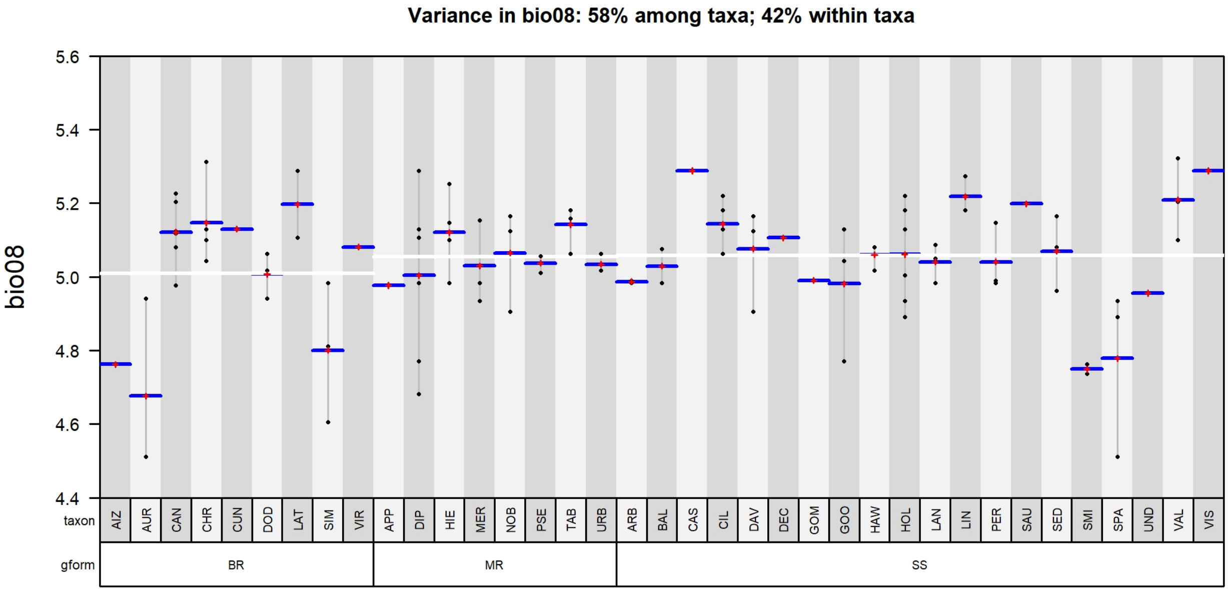

Supplementary Figure 19. Among and within taxa variance of BIO08 (mean temperature of the wettest quarter). BIO08 values are log-transformed. Each point represents one population; blue lines represent the taxon-mean values; white lines represent the growth form mean values.

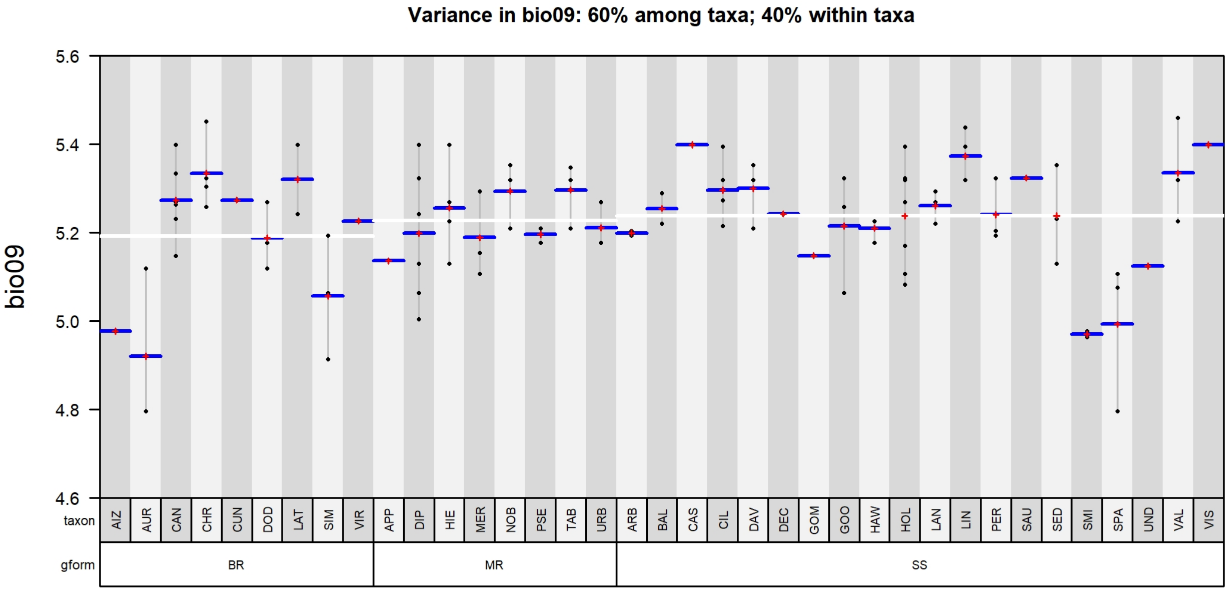

Supplementary Figure 20. Among and within taxa variance of BIO09 (mean temperature of the driest quarter). BIO09 values are log-transformed. Each point represents one population; blue lines represent the taxon-mean values; white lines represent the growth form mean values.

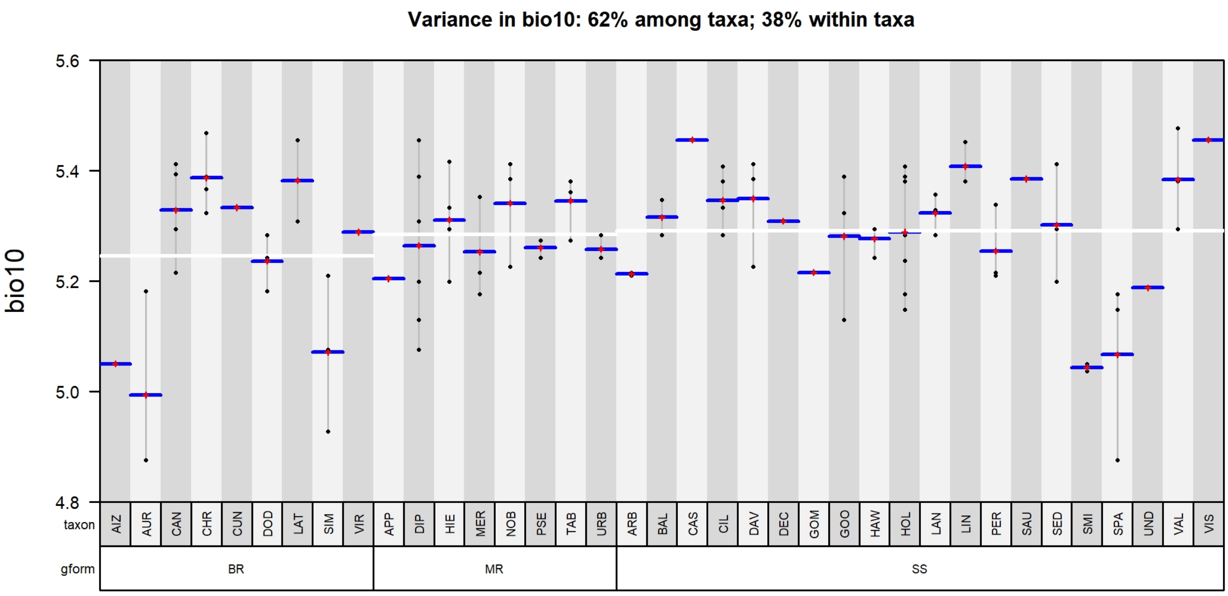

Supplementary Figure 21. Among and within taxa variance of BIO10 (mean temperature of the warmest quarter). BIO10 values are log-transformed. Each point represents one population; blue lines represent the taxon-mean values; white lines represent the growth form mean values.

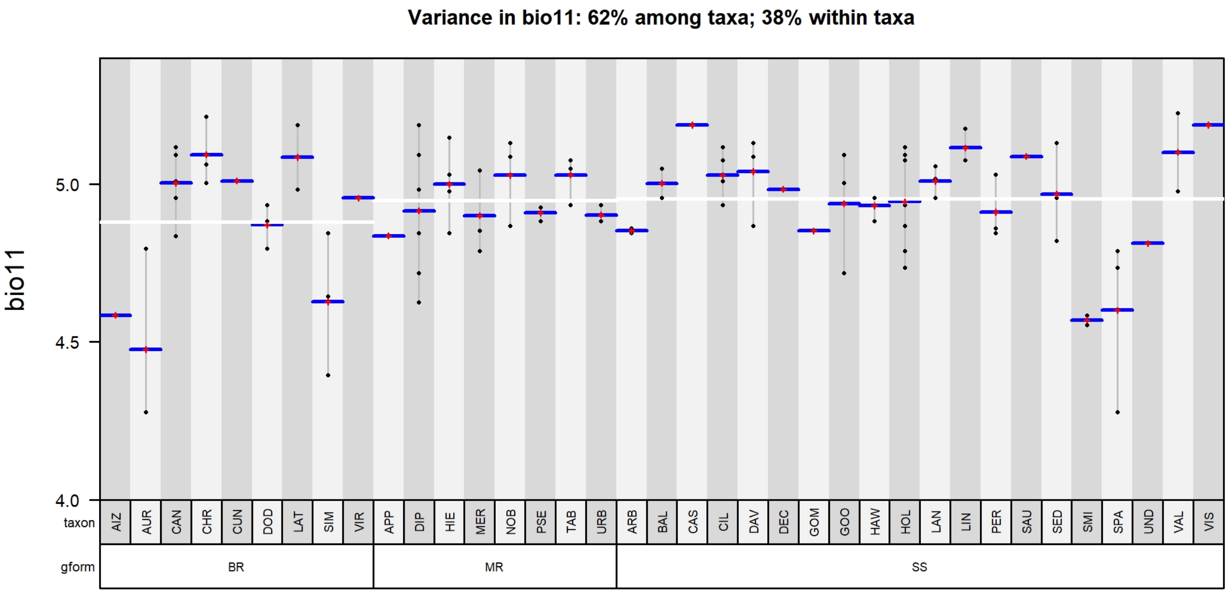

Supplementary Figure 22. Among and within taxa variance of BIO11 (mean temperature of the coldest quarter). BIO1 values are log-transformed. Each point represents one population; blue lines represent the taxon-mean values; white lines represent the growth form mean values.

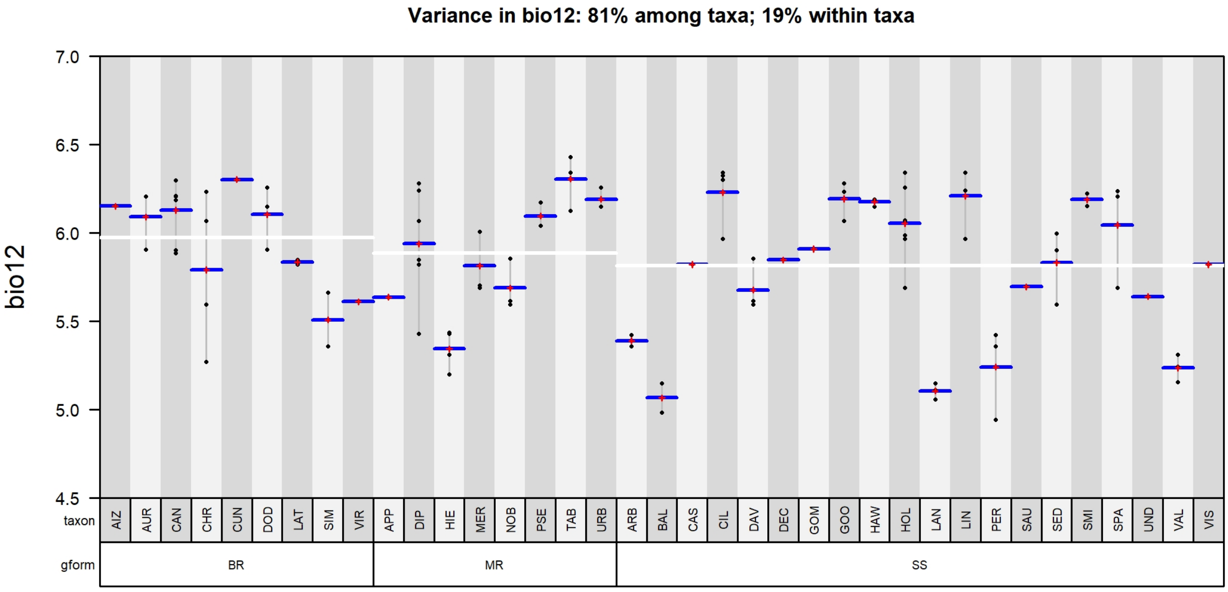

Supplementary Figure 23. Among and within taxa variance of BIO12 (annual precipitation). BIO12 values are log-transformed. Each point represents one population; blue lines represent the taxon-mean values; white lines represent the growth form mean values.

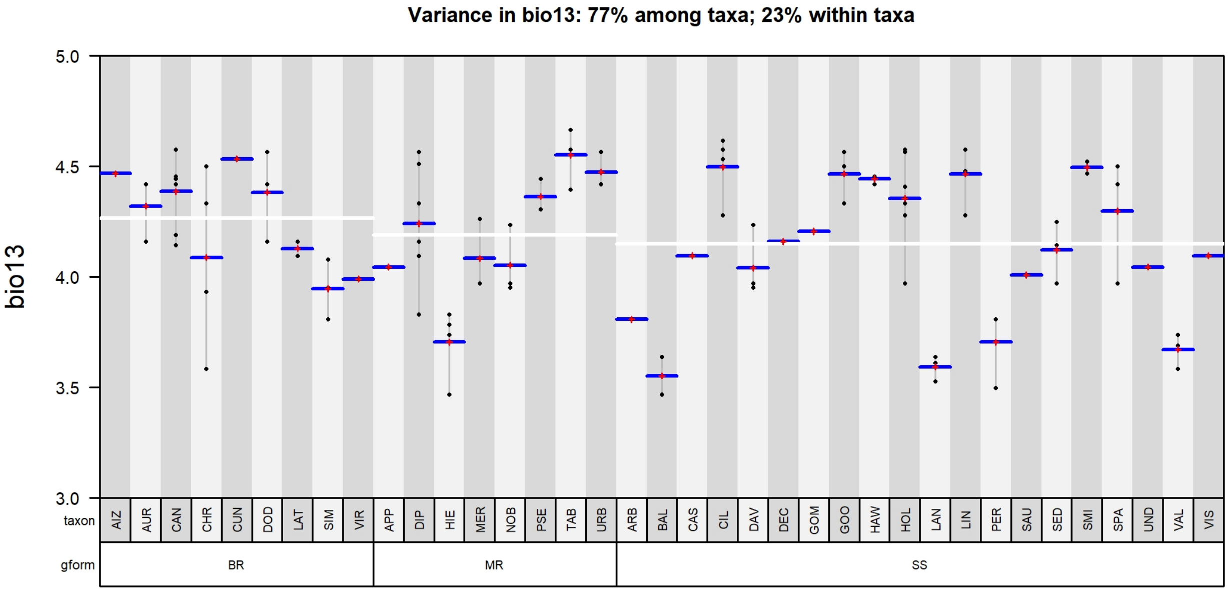

Supplementary Figure 24. Among and within taxa variance of BIO13 (precipitation of the wettest month). BIO13 values are log-transformed. Each point represents one population; blue lines represent the taxon-mean values; white lines represent the growth form mean values.

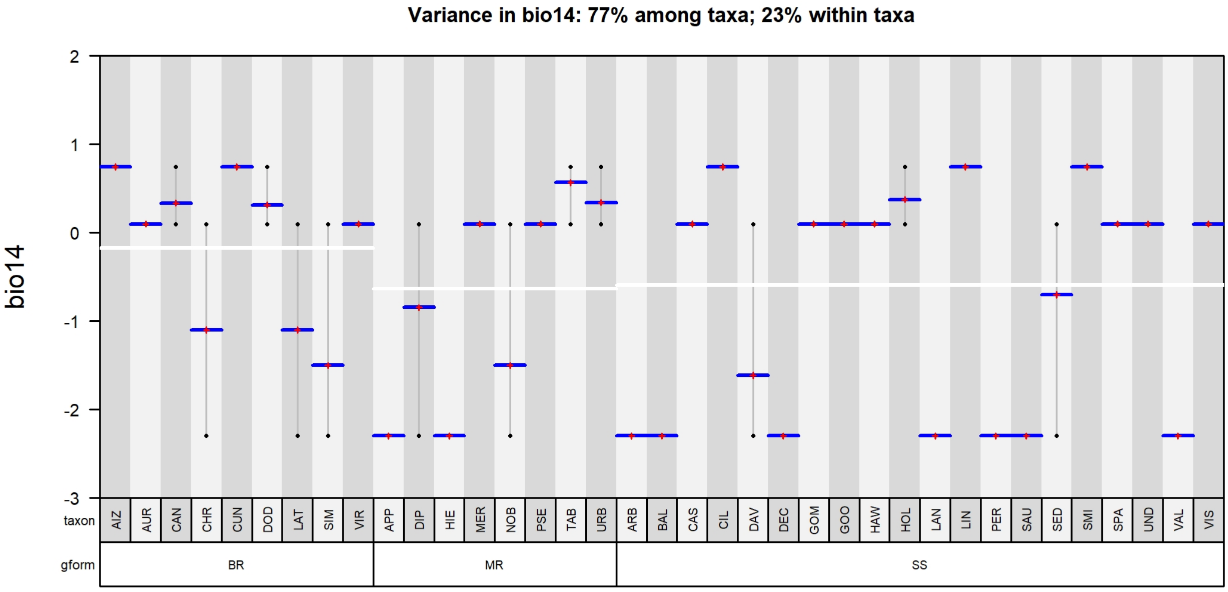

Supplementary Figure 25. Among and within taxa variance of BIO14 (precipitation of the driest month). BIO14 values are log-transformed. Each point represents one population; blue lines represent the taxon-mean values; white lines represent the growth form mean values.

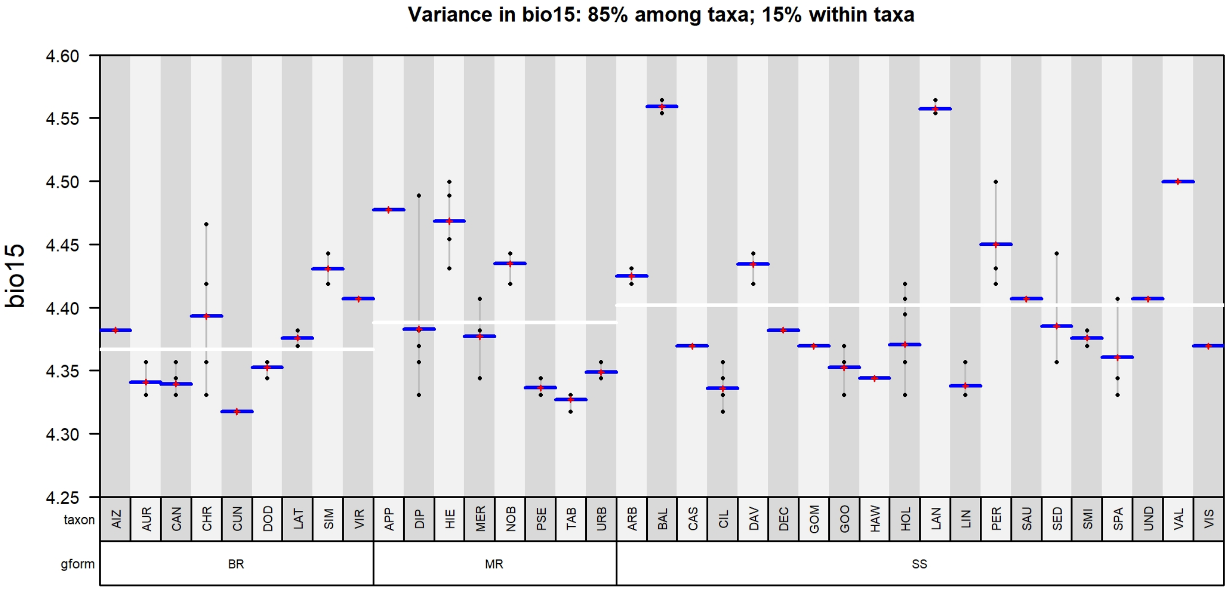

Supplementary Figure 26. Among and within taxa variance of BIO15 (precipitation seasonality). BIO15 values are log-transformed. Each point represents one population; blue lines represent the taxon-mean values; white lines represent the growth form mean values.

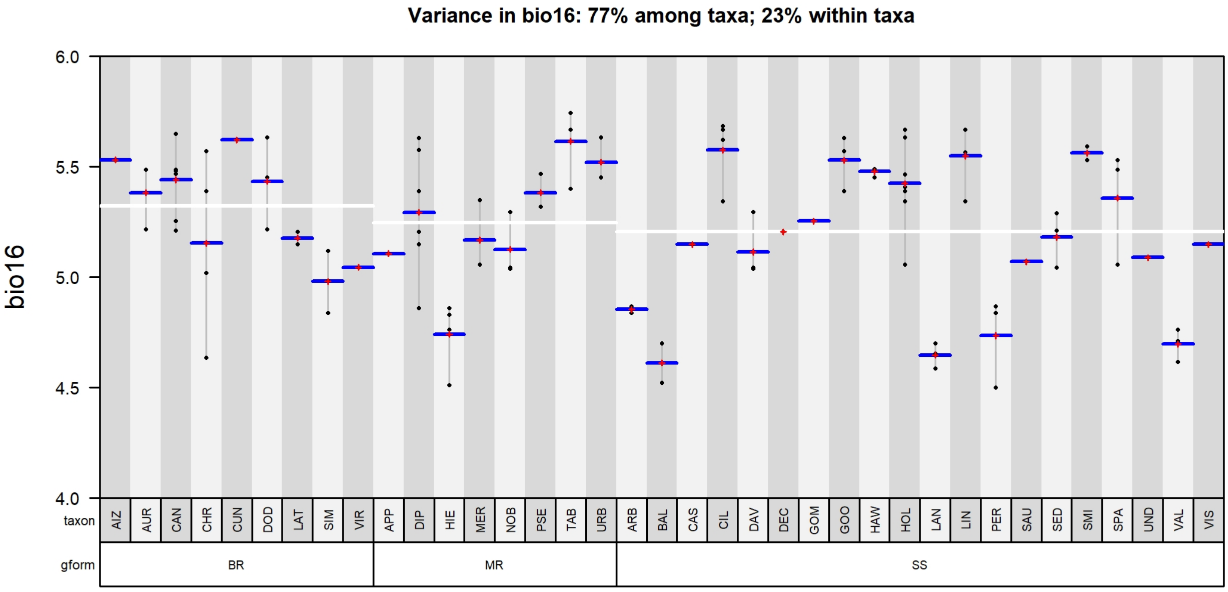

Supplementary Figure 27. Among and within taxa variance of BIO16 (precipitation of the wettest quarter). BIO16 values are log-transformed. Each point represents one population; blue lines represent the taxon-mean values; white lines represent the growth form mean values.

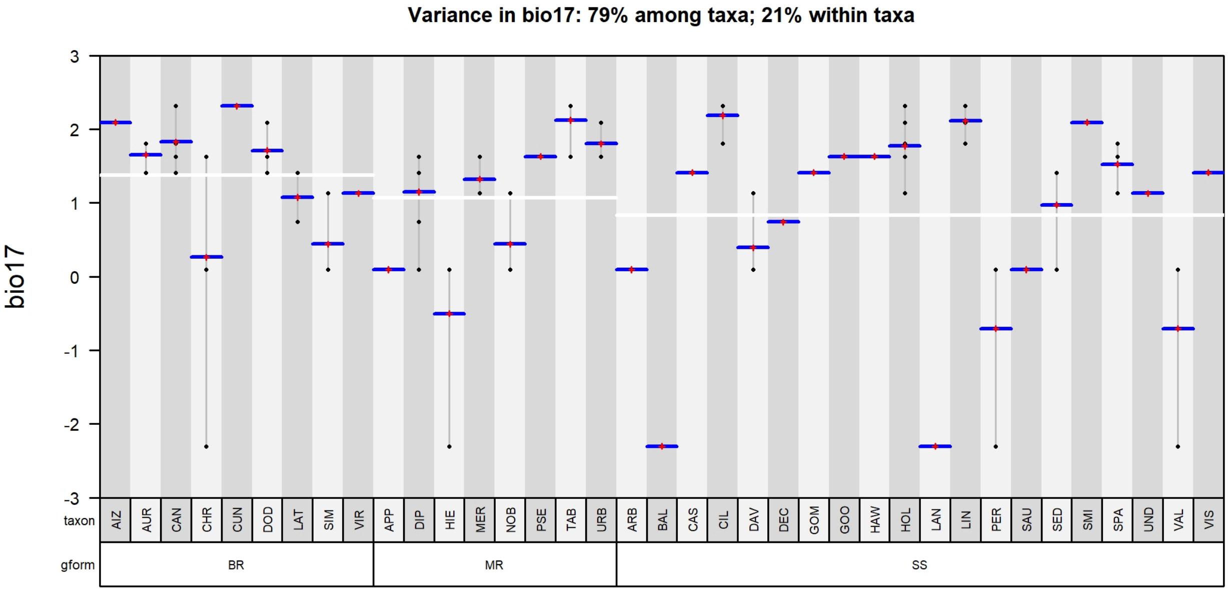

Supplementary Figure 28. Among and within taxa variance of BIO17 (precipitation of the driest quarter). BIO17 values are log-transformed. Each point represents one population; blue lines represent the taxon-mean values; white lines represent the growth form mean values.

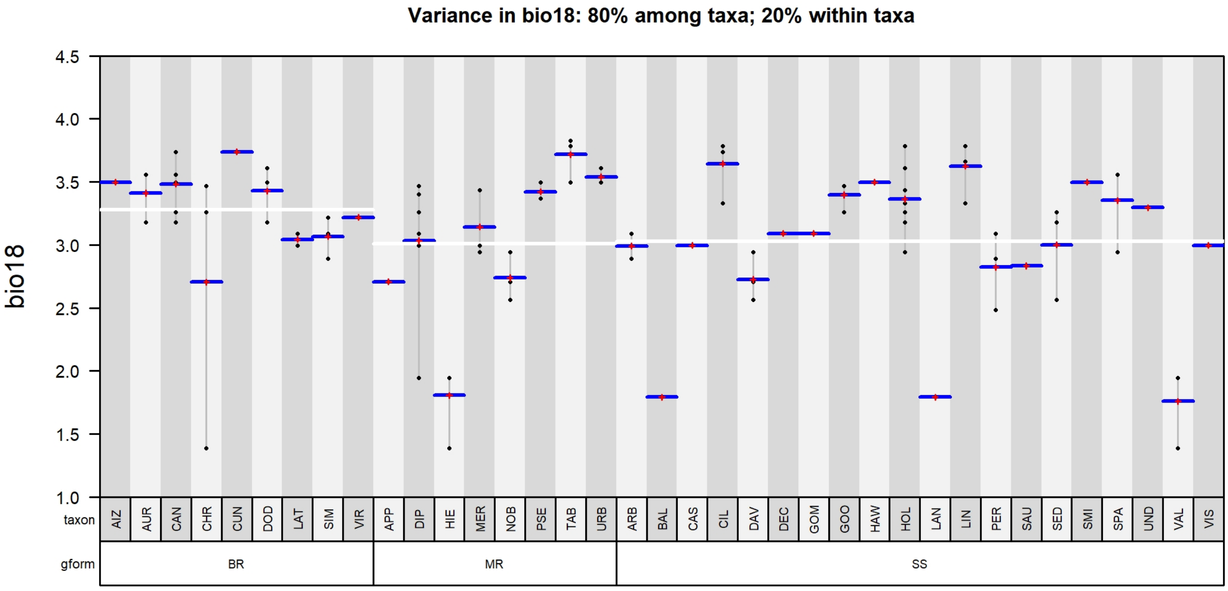

Supplementary Figure 29. Among and within taxa variance of BIO18 (precipitation of the warmest quarter). BIO18 values are log-transformed. Each point represents one population; blue lines represent the taxon-mean values; white lines represent the growth form mean values.

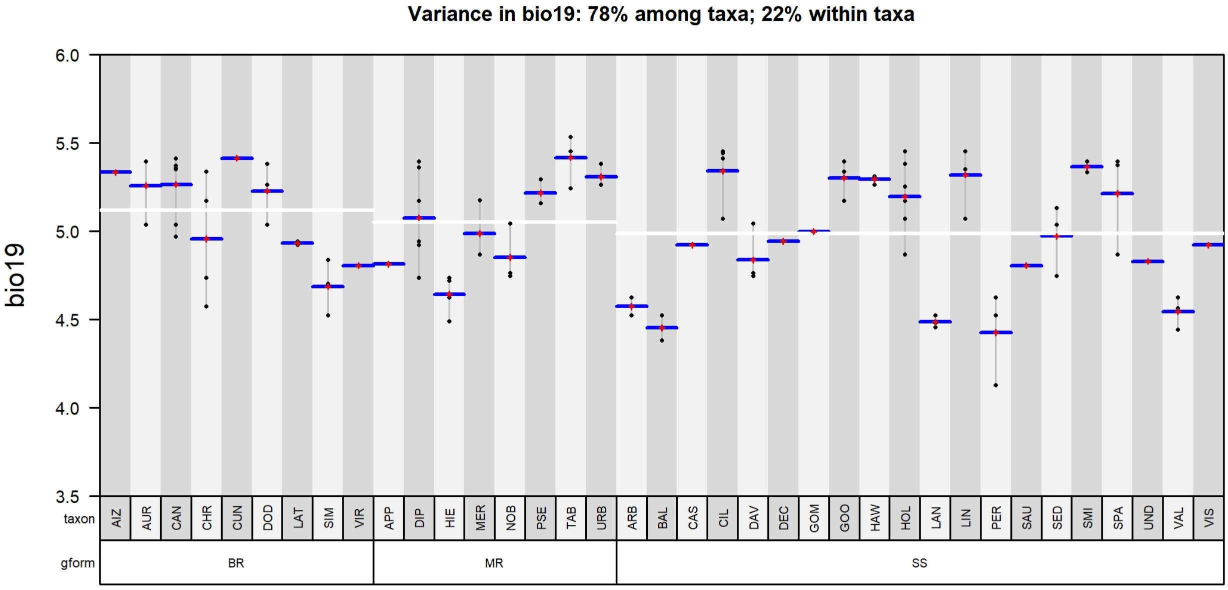

Supplementary Figure 30. Among and within taxa variance of BIO19 (precipitation of the coldest quarter). BIO19 values are log-transformed. Each point represents one population; blue lines represent the taxon-mean values; white lines represent the growth form mean values.

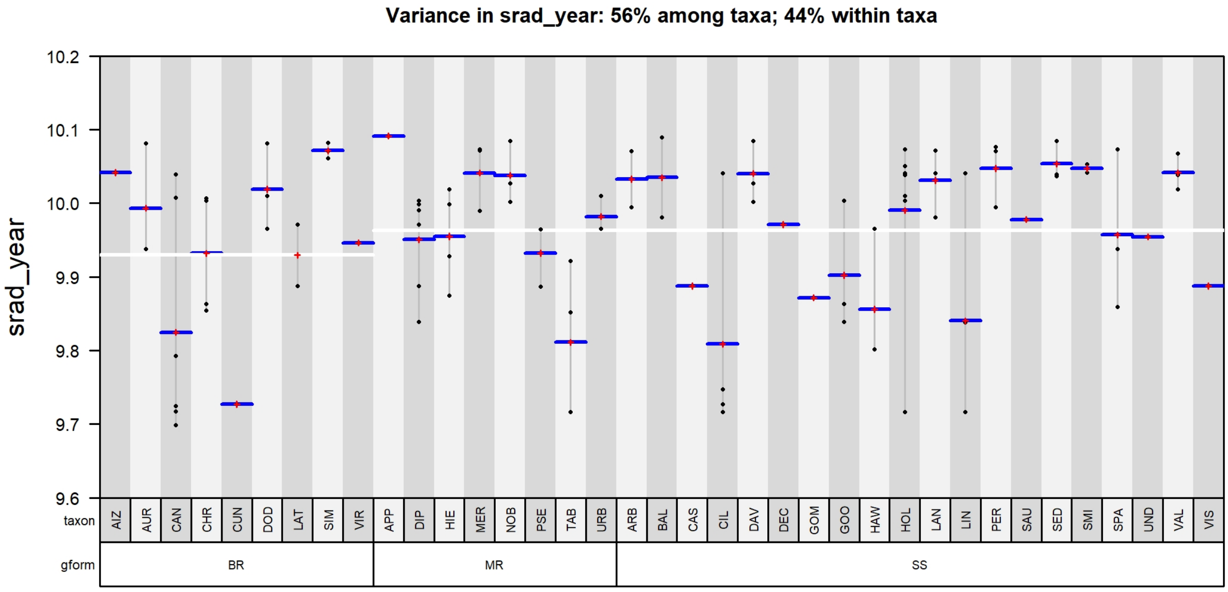

Supplementary Figure 31. Among and within taxa variance of solar radiation. Solar radiation values are log-transformed. Each point represents one population; blue lines represent the taxon-mean values; white lines represent the growth form mean values.

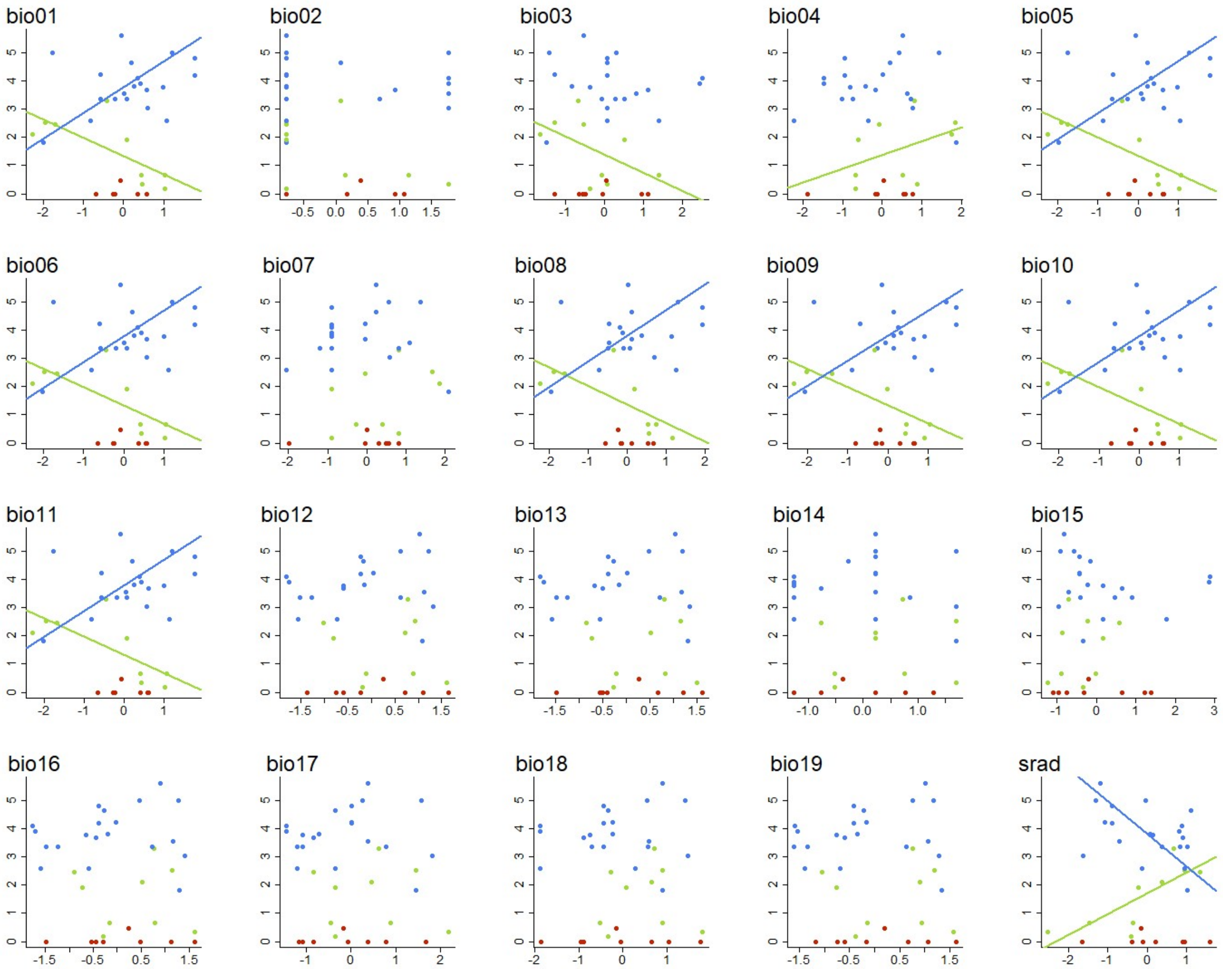

Supplementary Figure 32. Correlations between climatic variables and rosette number. Phylogenetic Generalized Least Square (PGLS) regressions of rosette number (rosette_no) on climatic variables (BIO01-BIO19 and solar radiation) among different growth forms. Rosette numbers are log-transformed; climatic variables are scaled. Each dot represents the mean of one taxon. Lines represent statistically significant regressions ($p < 0.05$). Colours indicate different growth forms: green – BR (branching rosettes), red – MR (monocarpic rosettes), blue – SS (shrubs or subshrubs).

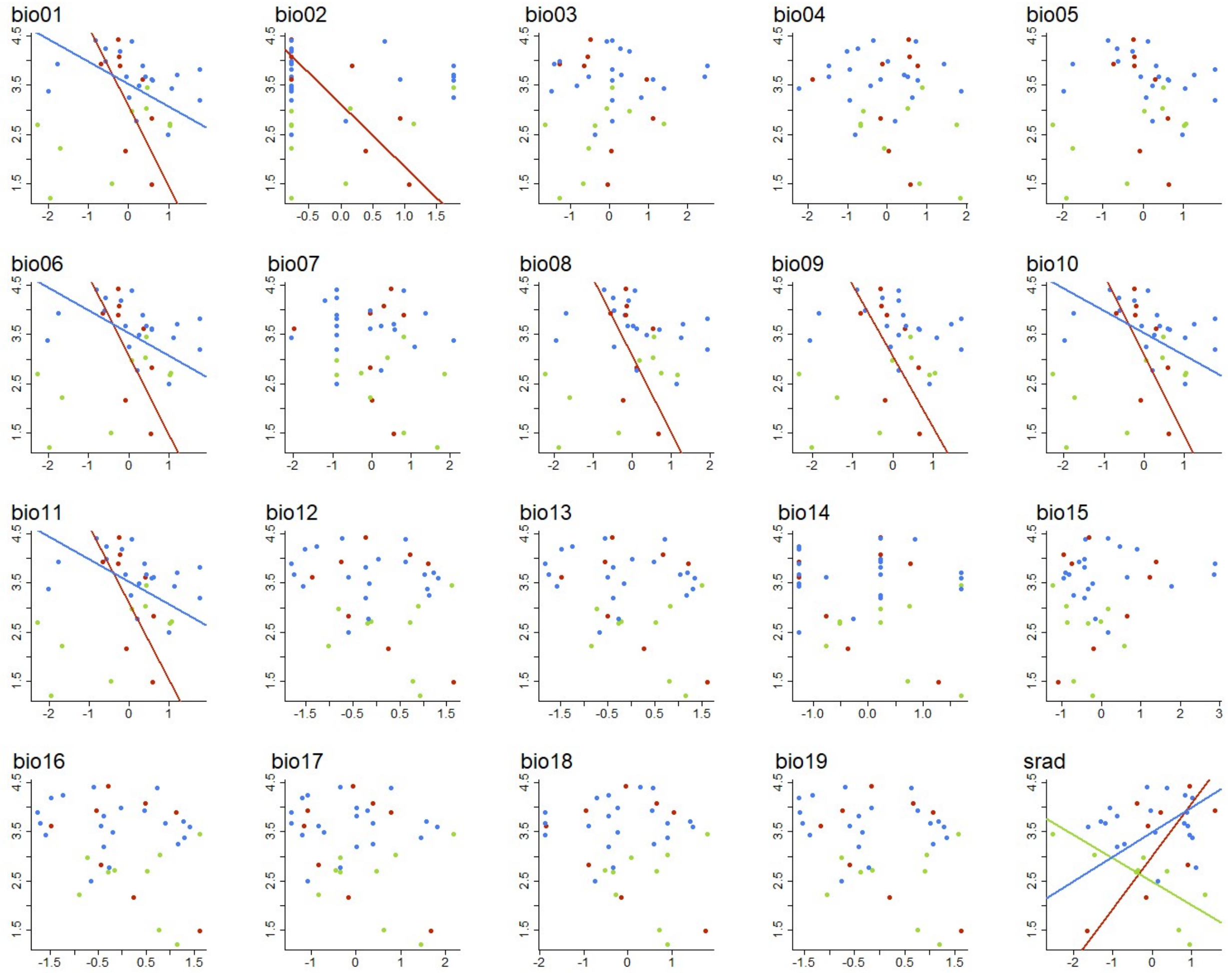

Supplementary Figure 33. Correlations between climatic variables and plant height. Phylogenetic Generalized Least Square (PGLS) regressions of plant height (height) on climatic variables (BIO01-BIO19 and solar radiation) among different growth forms. Plant height values are log-transformed; climatic variables are scaled. Each dot represents the mean of one taxon. Lines represent statistically significant regressions ($p < 0.05$). Colours indicate different growth forms: green – BR (branching rosettes), red – MR (monocarpic rosettes), blue – SS (shrubs or subshrubs).

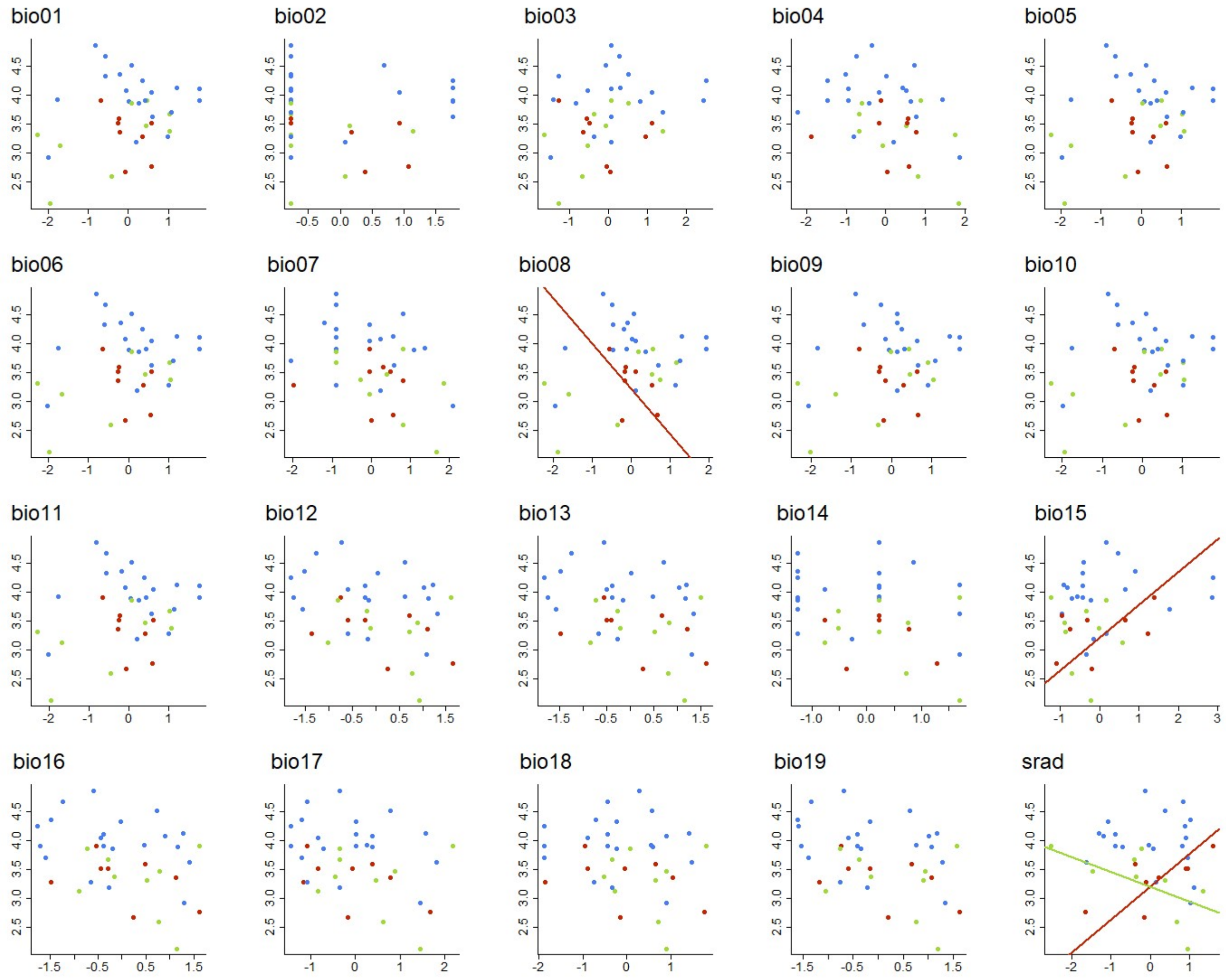

Supplementary Figure 34. Correlations between climatic variables and maximum plant diameter. Phylogenetic Generalized Least Square (PGLS) regressions of maximum plant diameter (DM) on climatic variables (BIO01-BIO19 and solar radiation) among different growth forms. Maximum plant diameter values are log-transformed; climatic variables are scaled. Each dot represents the mean of one taxon. Lines represent statistically significant regressions ($p < 0.05$). Colours indicate different growth forms: green – BR (branching rosettes), red – MR (monocarpic rosettes), blue – SS (shrubs or subshrubs).

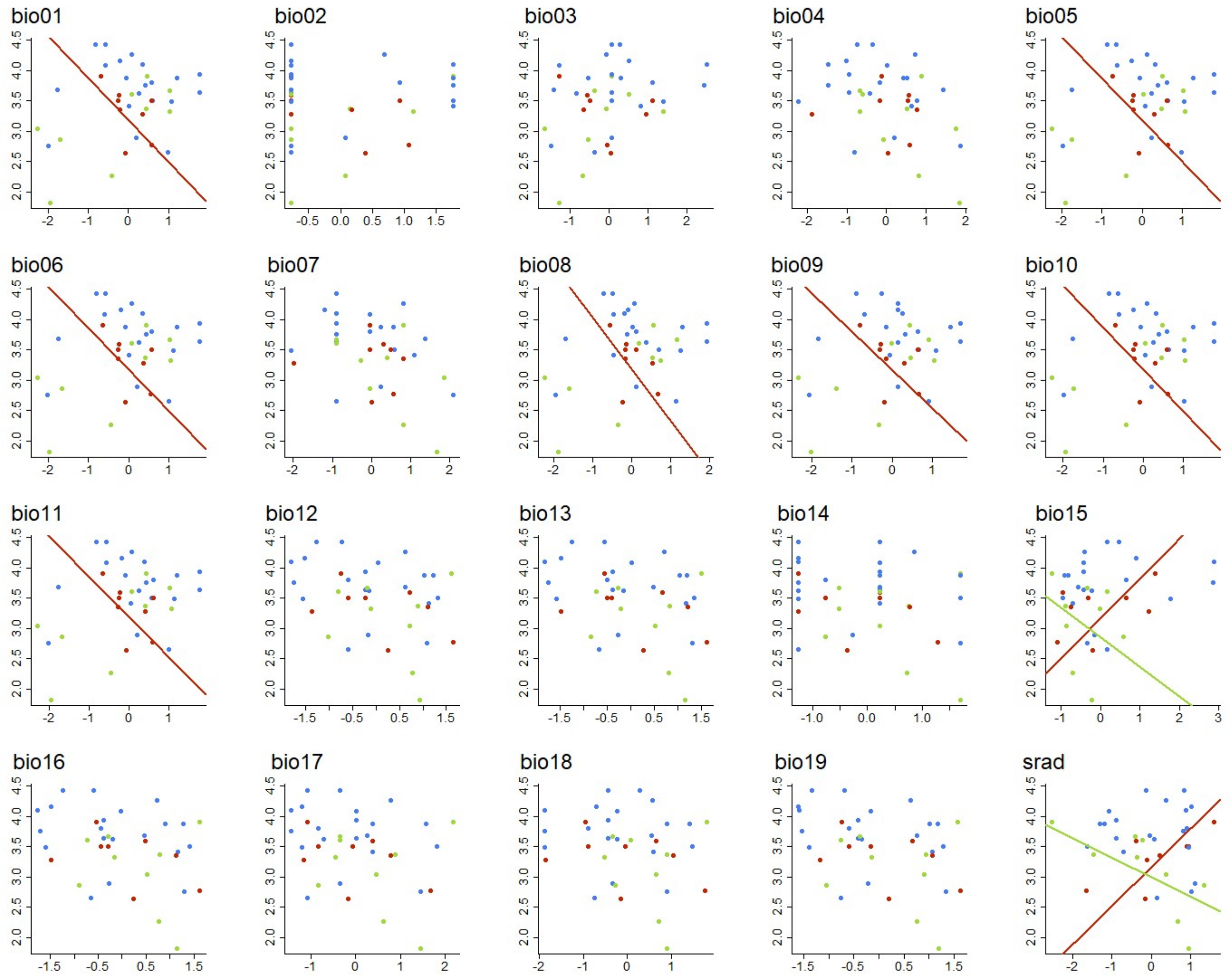

Supplementary Figure 35. Correlations between climatic variables and minimum plant diameter. Phylogenetic Generalized Least Square (PGLS) regressions of minimum plant diameter (Dm) on climatic variables (BIO01-BIO19 and solar radiation) among different growth forms. Minimum plant diameter values are log-transformed; climatic variables are scaled. Each dot represents the mean of one taxon Lines represent statistically significant regressions ($p < 0.05$). Colours indicate different growth forms: green – BR (branching rosettes), red – MR (monocarpic rosettes), blue – SS (shrubs or subshrubs).

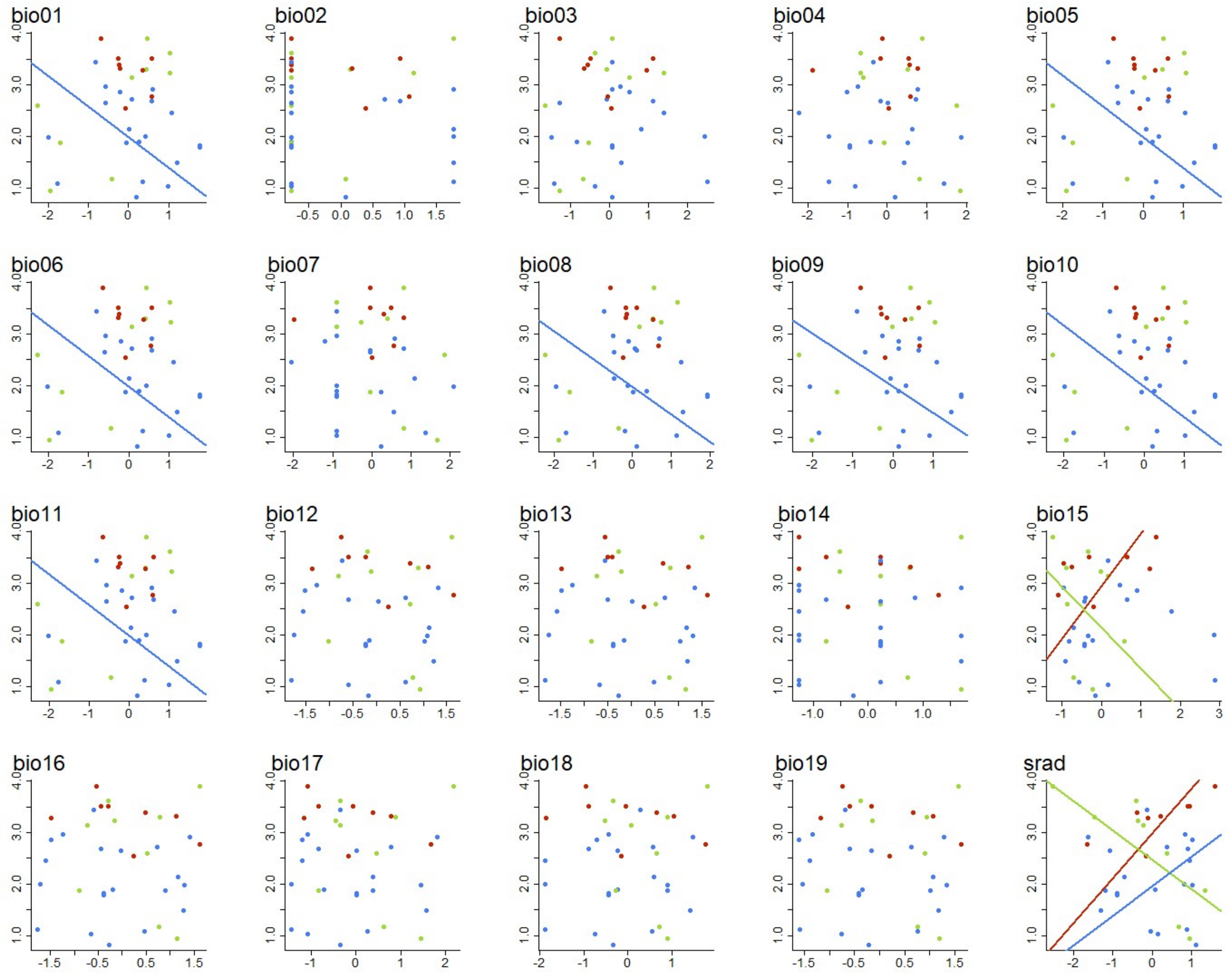

Supplementary Figure 36. Correlations between climatic variables and rosette diameter. Phylogenetic Generalized Least Square (PGLS) regressions of rosette diameter (meanrosdiam) on climatic variables (BIO01-BIO19 and solar radiation) among different growth forms. Rosette diameter values are log-transformed; climatic variables are scaled. Each dot represents the mean of one taxon. Lines represent statistically significant regressions ($p < 0.05$). Colours indicate different growth forms: green – BR (branching rosettes), red – MR (monocarpic rosettes), blue – SS (shrubs or subshrubs).

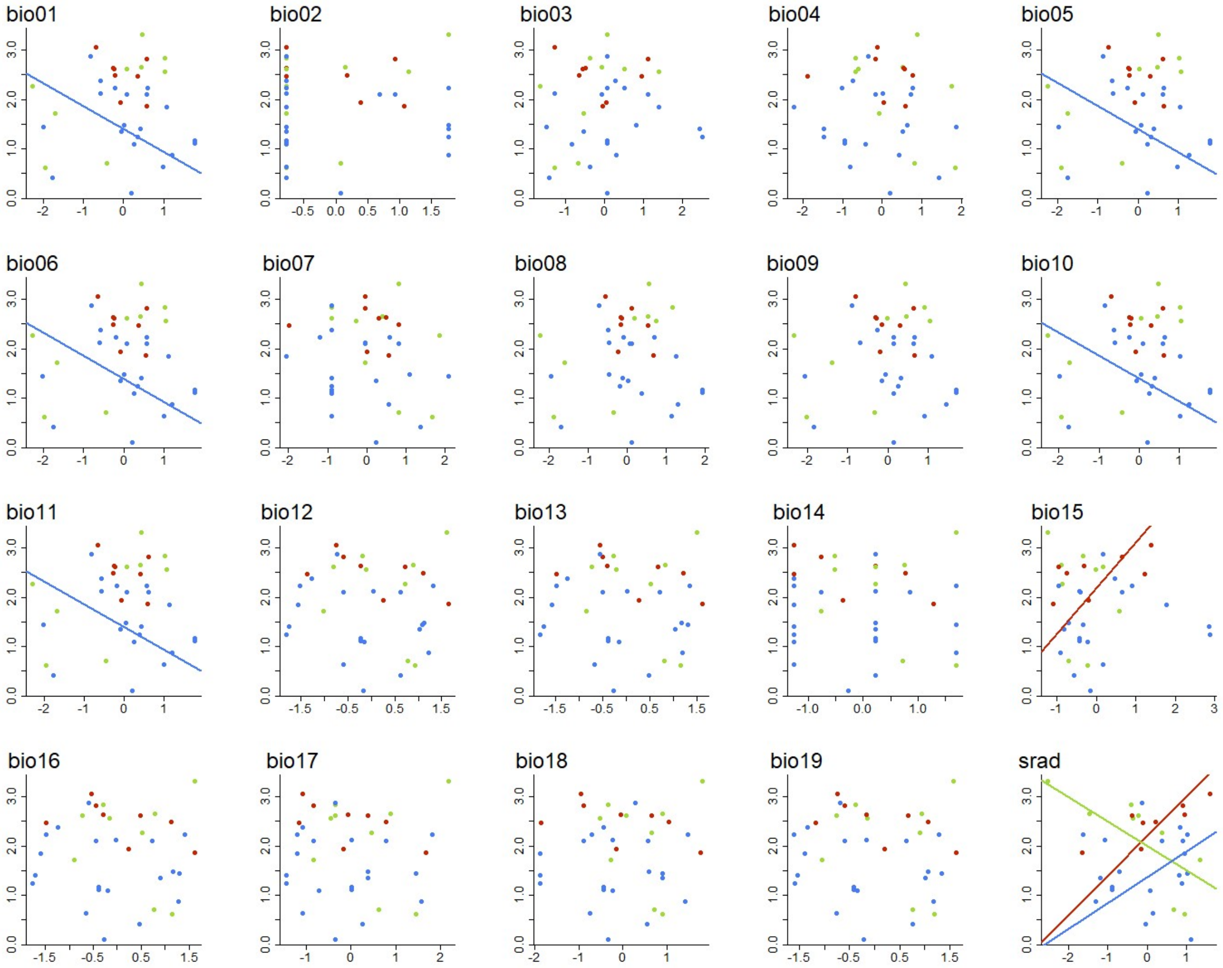

Supplementary Figure 37. Correlations between climatic variables and leaf length. Phylogenetic Generalized Least Square (PGLS) regressions of leaf length (length) on climatic variables (BIO01-BIO19 and solar radiation) among different growth forms. Leaf length values are log-transformed; climatic variables are scaled. Each dot represents the mean of one taxon. Lines represent statistically significant regressions ($p < 0.05$). Colours indicate different growth forms: green – BR (branching rosettes), red – MR (monocarpic rosettes), blue – SS (shrubs or subshrubs).

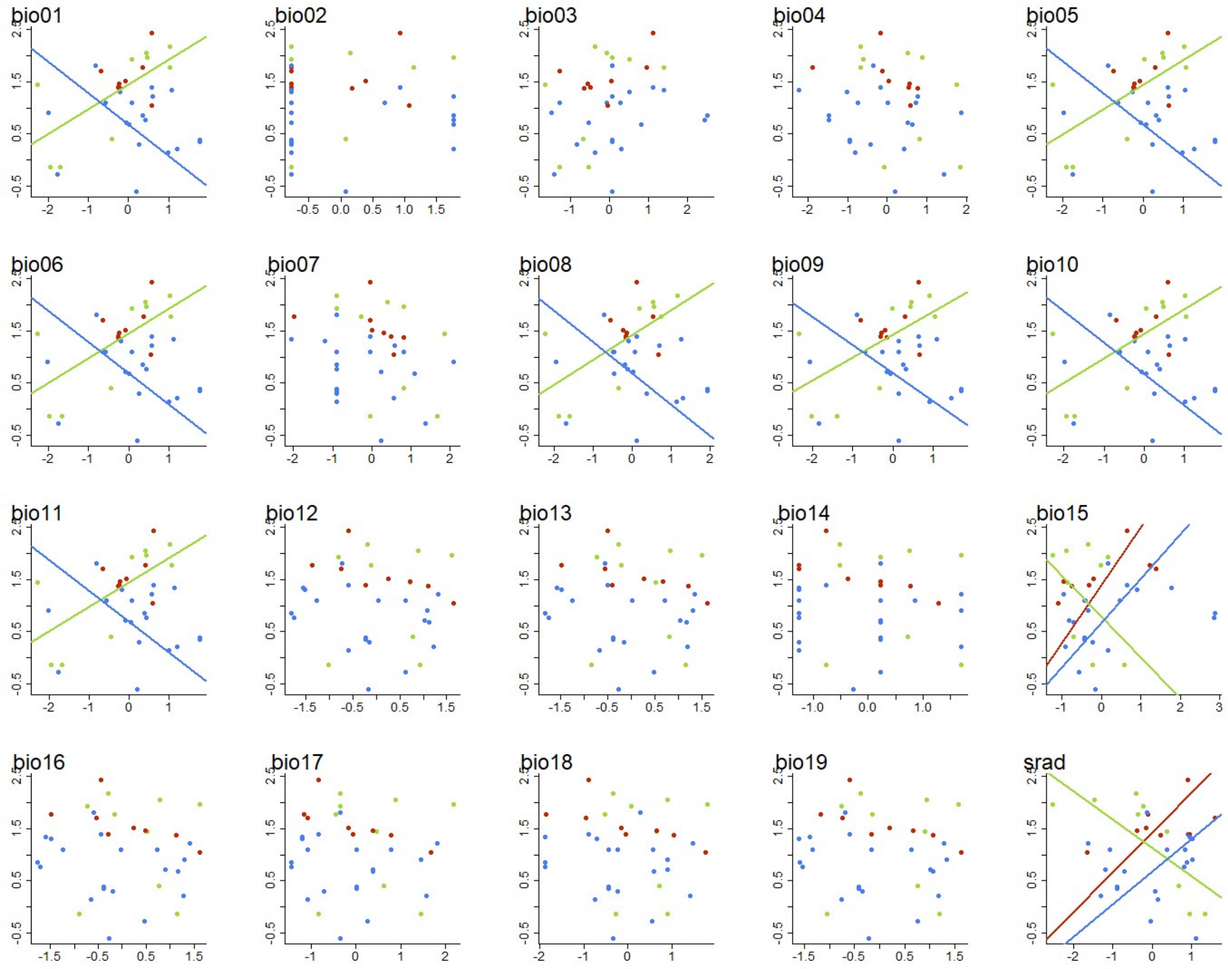

Supplementary Figure 38. Correlations between climatic variables and leaf width. Phylogenetic Generalized Least Square (PGLS) regressions of leaf width (width) on climatic variables (BIO01-BIO19 and solar radiation) among different growth forms. Leaf width values are log-transformed; climatic variables are scaled. Each dot represents the mean of one species. Lines represent statistically significant regressions ($p < 0.05$). Colours indicate different growth forms: green – BR (branching rosettes), red – MR (monocarpic rosettes), blue – SS (shrubs or subshrubs).

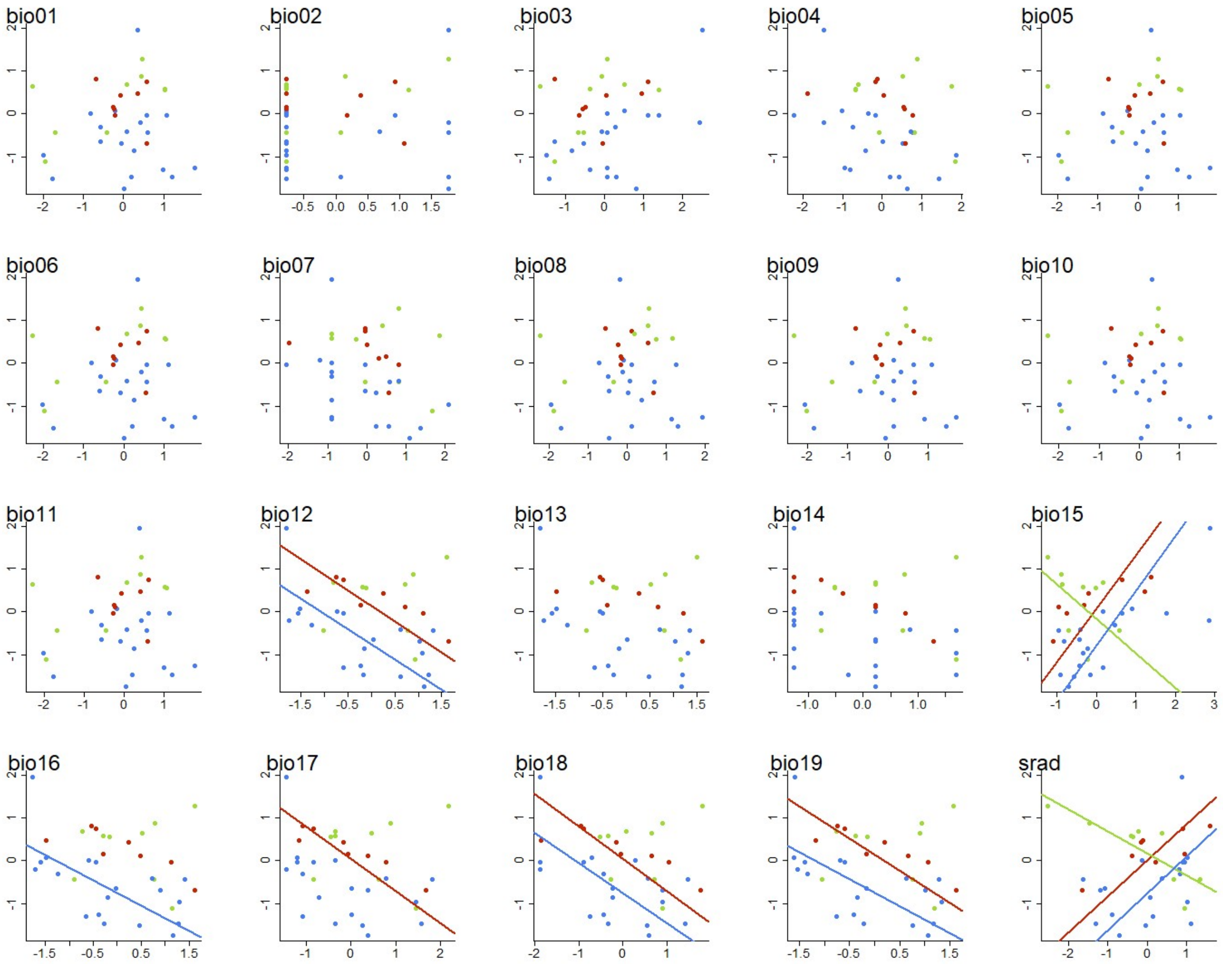

Supplementary Figure 39. Correlations between climatic variables and petiole width. Phylogenetic Generalized Least Square (PGLS) regressions of petiole width (widthbase) on climatic variables (BIO01-BIO19 and solar radiation) among different growth forms. Petiole width values are log-transformed; climatic variables are scaled. Each dot represents the mean of one taxon Lines represent statistically significant regressions ($p < 0.05$). Colours indicate different growth forms: green – BR (branching rosettes), red – MR (monocarpic rosettes), blue – SS (shrubs or subshrubs).

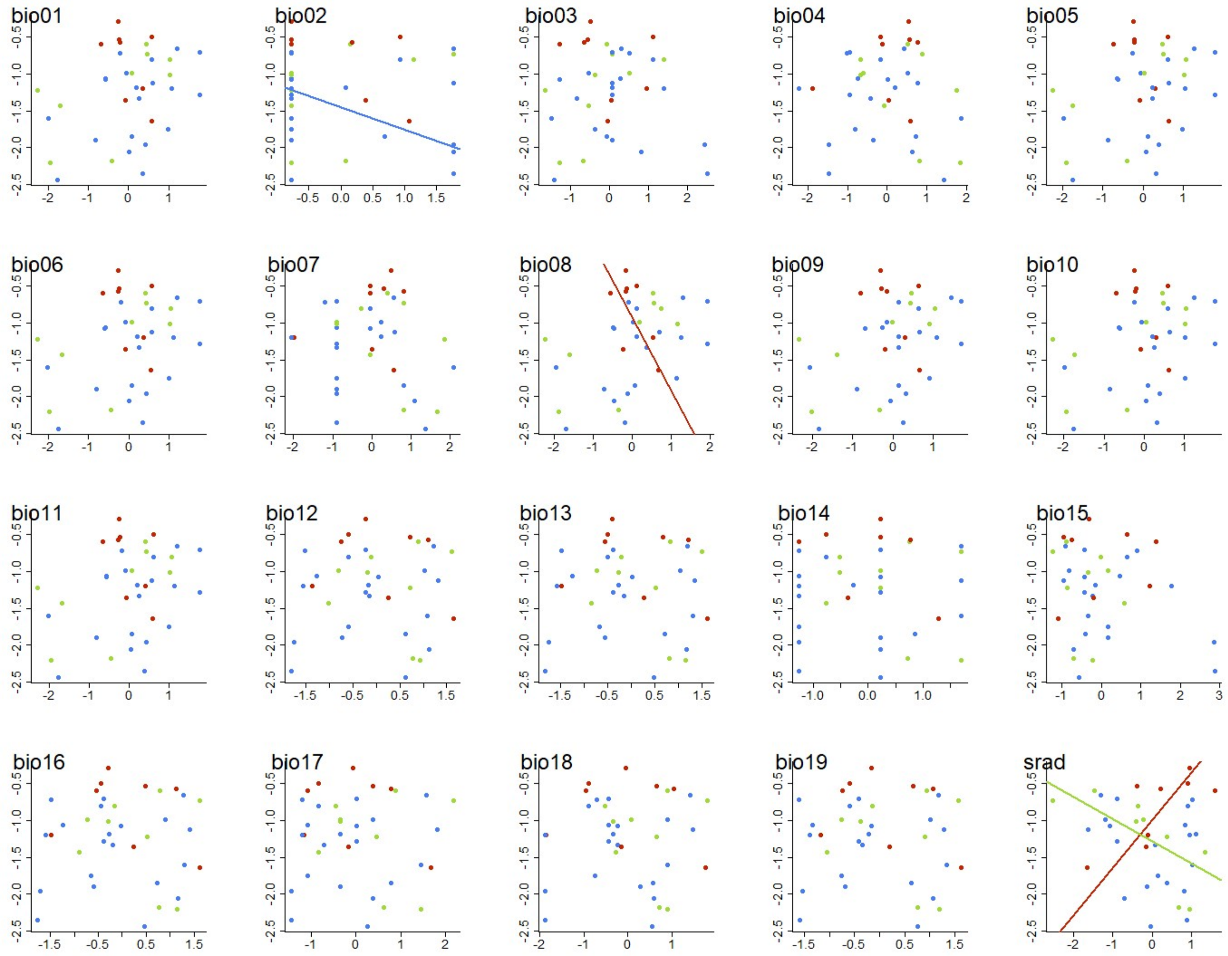

Supplementary Figure 40. Correlations between climatic variables and leaf thickness. Phylogenetic Generalized Least Square (PGLS) regressions of leaf thickness (thickness) on climatic variables (BIO01-BIO19 and solar radiation) among different growth forms. Leaf thickness values are log-transformed; climatic variables are scaled. Each dot represents the mean of one taxon. Lines represent statistically significant regressions ($p < 0.05$). Colours indicate different growth forms: green – BR (branching rosettes), red – MR (monocarpic rosettes), blue – SS (shrubs or subshrubs).

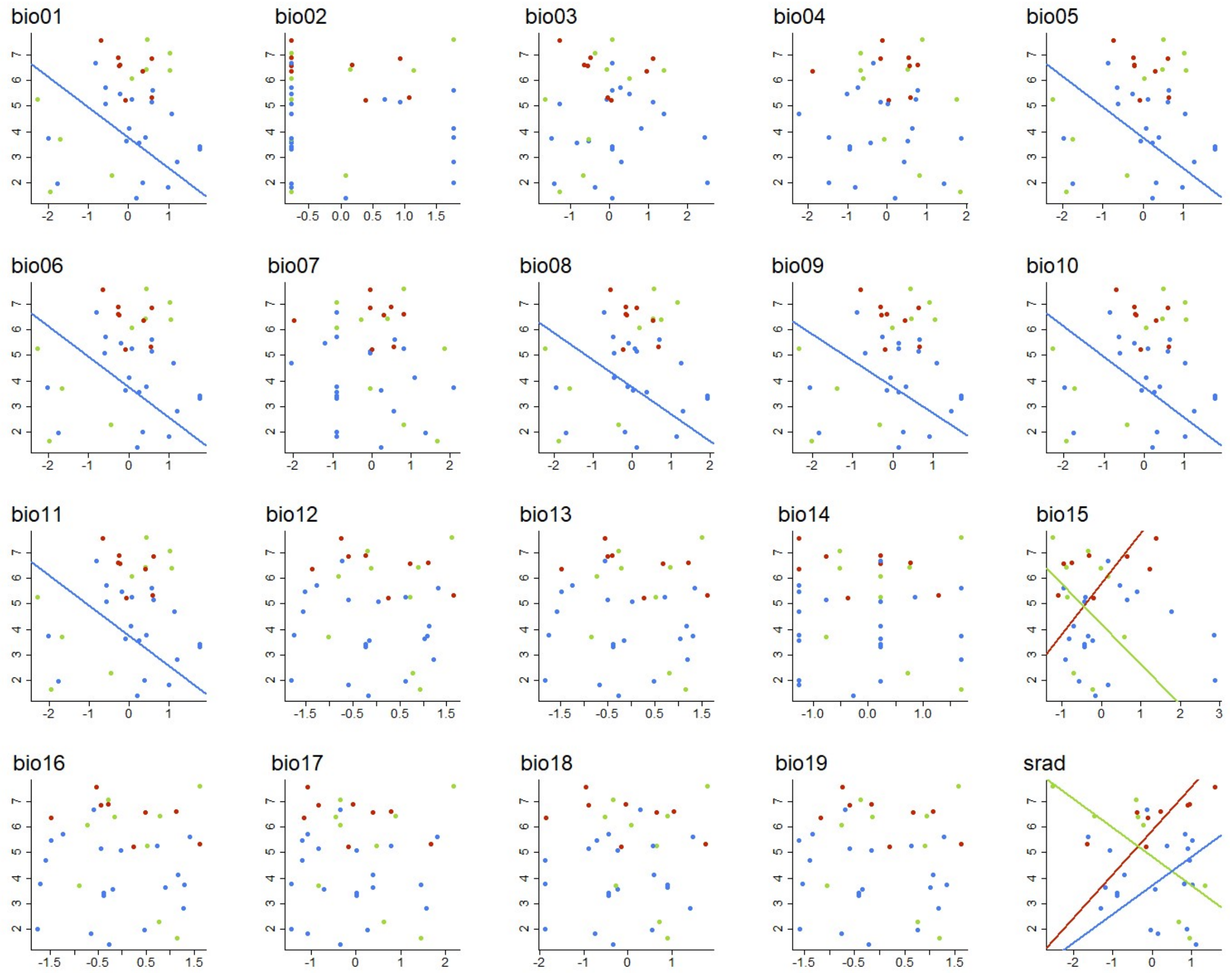

Supplementary Figure 41. Correlations between climatic variables and rosette area. Phylogenetic Generalized Least Square (PGLS) regressions of rosette area (ros_area) on climatic variables (BIO01-BIO19 and solar radiation) among different growth forms. Rosette area values are log-transformed; climatic variables are scaled. Each dot represents the mean of one species. Lines represent statistically significant regressions ($p < 0.05$). Colours indicate different growth forms: green – BR (branching rosettes), red – MR (monocarpic rosettes), blue – SS (shrubs or subshrubs).

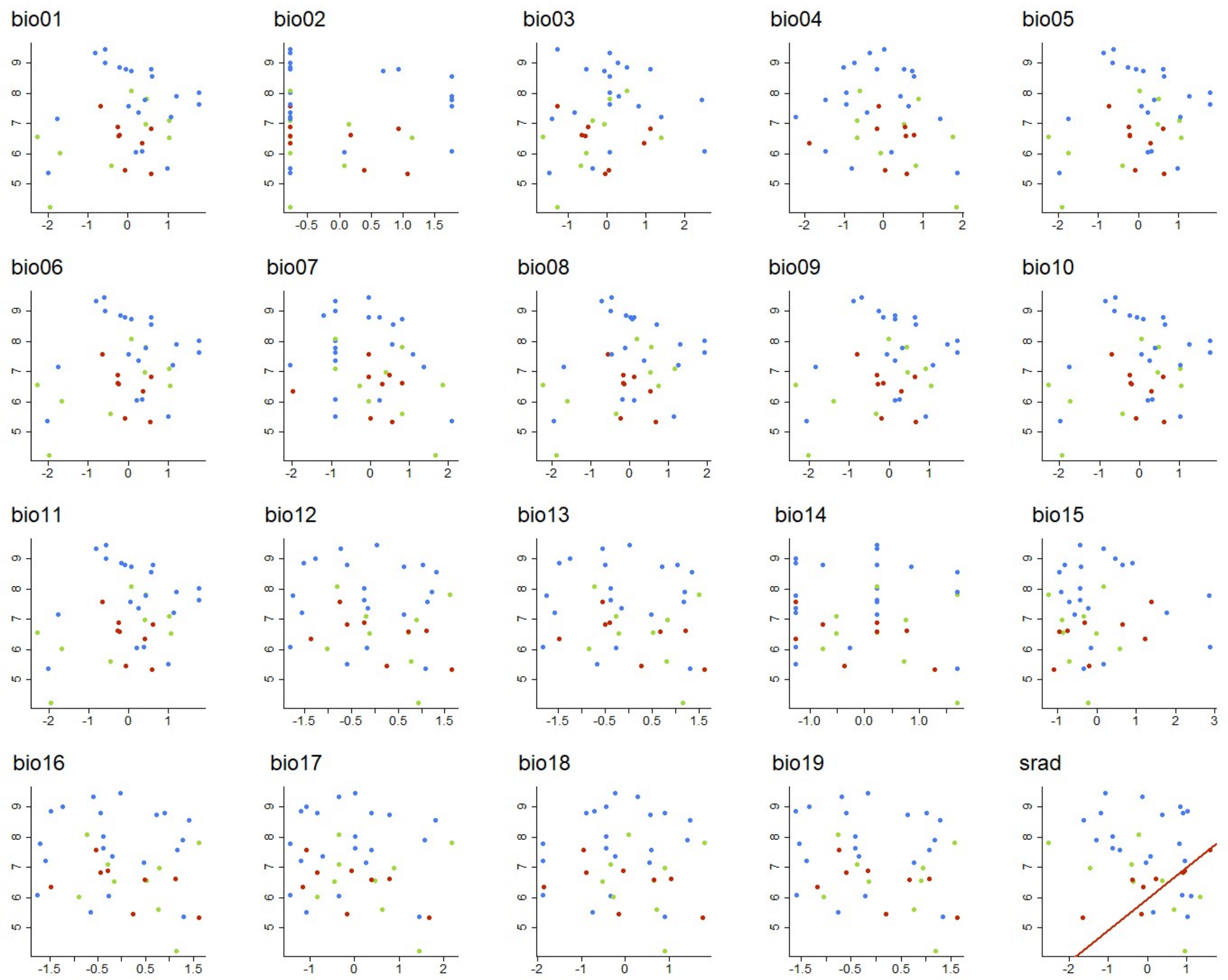

Supplementary Figure 42. Correlations between climatic variables and photosynthetic area. Phylogenetic Generalized Least Square (PGLS) regressions of photosynthetic area (photo_area) on climatic variables (BIO01-BIO19 and solar radiation) among different growth forms. Photosynthetic area values are log-transformed; climatic variables are scaled. Each dot represents the mean of one taxon. Lines represent statistically significant regressions ($p < 0.05$). Colours indicate different growth forms: green – BR (branching rosettes), red – MR (monocarpic rosettes), blue – SS (shrubs or subshrubs).
